# Smart Sensor Systems for Wearable Electronic Devices

**DOI:** 10.3390/polym9080303

**Published:** 2017-07-25

**Authors:** Byeong Wan An, Jung Hwal Shin, So-Yun Kim, Joohee Kim, Sangyoon Ji, Jihun Park, Youngjin Lee, Jiuk Jang, Young-Geun Park, Eunjin Cho, Subin Jo, Jang-Ung Park

**Affiliations:** School of Materials Science and Engineering, Wearable Electronics Research Group, Ulsan National Institute of Science and Technology (UNIST), Ulsan 44919, Korea; zcartom@unist.ac.kr (B.W.A.); shinjunghwal@unist.ac.kr (J.H.S.); syk0128@unist.ac.kr (S.-Y.K.); joohee710610@unist.ac.kr (J.K.); qweasrd@unist.ac.kr (S.J.); jhpark1005@unist.ac.kr (J.P.); yjlee0411@unist.ac.kr (Y.L.); dnr3146@unist.ac.kr (J.J.); younggeun@unist.ac.kr (Y.-G.P.); eunjincho@unist.ac.kr (E.C.); subin@unist.ac.kr (S.J.)

**Keywords:** smart sensor, wearable electronics, stretchable electronics, wireless sensor, healthcare

## Abstract

Wearable human interaction devices are technologies with various applications for improving human comfort, convenience and security and for monitoring health conditions. Healthcare monitoring includes caring for the welfare of every person, which includes early diagnosis of diseases, real-time monitoring of the effects of treatment, therapy, and the general monitoring of the conditions of people’s health. As a result, wearable electronic devices are receiving greater attention because of their facile interaction with the human body, such as monitoring heart rate, wrist pulse, motion, blood pressure, intraocular pressure, and other health-related conditions. In this paper, various smart sensors and wireless systems are reviewed, the current state of research related to such systems is reported, and their detection mechanisms are compared. Our focus was limited to wearable and attachable sensors. Section 1 presents the various smart sensors. In Section 2, we describe multiplexed sensors that can monitor several physiological signals simultaneously. Section 3 provides a discussion about short-range wireless systems including bluetooth, near field communication (NFC), and resonance antenna systems for wearable electronic devices.

## 1. Introduction

In recent years, the average age of the world’s population has been increasing, and healthcare monitoring devices have received a lot of attention because they improve the length and quality of people’s lives [1]. Healthcare monitoring includes caring for the welfare of every person, which includes early diagnosis of diseases, real-time monitoring of the effects of treatment, therapy, and the general monitoring of the conditions of people’s health. As a result, wearable electronic devices are receiving greater attention because of their facile interaction with the human body, such as monitoring heart rate, wrist pulse, motion, blood pressure, intraocular pressure, and other health-related conditions [2,3,4,5,6,7,8,9]. Unlike conventional health-monitoring systems (e.g., blood pressure meters), wearable electronic devices are portable, wearable, and they provide real-time, continuous, recorded data related to complex health conditions in a timely manner. Furthermore, in order to maximize the portable and wearable advantages of the wearable devices, energy harvesting devices were integrated instead of rigid and bulk batteries [10,11]. These characteristics of wearable electronic devices can improve the users’ or patients’ compliance with medical instructions and medication schedules. Since the wearable devices are wireless, the information they gather can be sent to a central node, such as a cell phone or a microcontroller board that can transmit the information to a medical center and display it on a screen.

In general, wearable electronic systems consist of smart sensors, wearable materials, actuators, power supplies, wireless communication modules and links, control and processing units, an interface for the user, software, and advanced algorithms for extracting data and making decisions. Thus, the systems can monitor the patient’s physiological information, such as temperature, blood pressure, strain the patient may be experiencing, and the concentrations of gas, various ions, and biomolecules in the bloodstream. To be used in wearable electronic systems, the smart sensors, which are composed of flexible substrates and have embedded, conducting electrodes, must be ultrathin, low modulus, light weight, highly flexible, and stretchable.

To fabricate the smart sensors having these requirements, various studies using hybrid structures [12,13,14], hybrid materials [15,16,17,18,19,20,21,22], multi-dimensional carbon nanofibers [23], and nanomaterial-based electrodes [24] have been introduced. In addition, electrohydrodynamic (EHD) inkjet printing and three dimensional (3D) printing methods are widely used to make the electrodes with high-resolution and high-performance [16,25,26,27,28].

Beyond the concept of wearable appliances, sensor is rapidly evolving toward a new paradigm of deformable electronics, where sensors can be wearable into various human body parts. Wearable sensor is the most sophisticated form of deformable sensors in that it involves state-of-the-art technologies developed through the collaboration of various disciplines, ranging from materials science to electrical and mechanical engineering. As well described in some excellent previous reports in Table 1, substrate, electrode, and active materials are important building blocks of wearable sensor. The most important part for the flexibility or stretchability of the wearable sensor is the substrate. In order to realize a flexible sensor, flexible substrates such as polyethylene terephthalate (PET) and polyimide (PI) films are used [12,18,29]. PET film has good flexibility (flexural modulus: 8.3–14 GPa, Young’s modulus: 3.5–11 GPa); however, low glass transition temperature (*T_g_*: ≈100 °C) is not suitable for high temperature fabrication. PI film show good thermal stability (*T_g_*: ≈300 °C) due to strong imide bonding, so high temperature fabrication can be possible. Also, PI has good flexibility as well as PET (flexural modulus: 3.9–19 GPa, Young’s modulus: 3.7–20 GPa). Basic PI film has yellowish-brown color. However, transparent and colorless PI films have been developed for use in transparent devices and wearable electronic in recent years. In addition, clothing type sensors were fabricated using fabric-type of PET substrate to demonstrate wearable sensors [30].

Stretchable and wearable sensors essentially require stretchable substrates. Silicon-based elastomers such as polydimethylsiloxane (PDMS) and Ecoflex are selected as stretchable substrates in numerous researches of wearable sensors, which are formed by the polymerization of siloxane monomers with platinum catalysts [3,31,32,33,34]. PDMS shows 4.8 MPa of Young’s modulus and 420% of elongation limit. Ecoflex shows 900% of elongation limit. These elastomers polymer chain entanglements with low intermolecular force, thus they show good stretchability. Besides the silicon-based elastomers, parylene is also used for the substrate of thin-film wearable sensors because of its strength and intermediate flexibility (Young’s modulus ≈ 4 GPa). In addition, parylene is known to be biocompatible in many reports [35].

Electrode parts are also important parts of wearable sensors. Substrate parts mostly use polymer-based materials, but in the case of electrodes, metal-based electrodes are used because high-conductivity is important. Thin-metal is used to form flexible interconnects or serpentine-structure metal to increase stretchablility [3,36,37,38]. As alternative approaches to achieving the stretchable interconnect, nanowire-based random network [12,18,31,32] and liquid metal. Liquid metal exhibits metal-like conductivity at room temperature, but due to the nature of the liquid, the failure does not occur under strain, and thus highly stretchable interconnects can be achieved.

Various materials can be used as the active material of the wearable sensor according to the type and purpose of the sensor. Strain or pressure sensor use a strain sensitive resistor or piezoelectric material that changes resistance according to strain or pressure. Chemical sensors also use various active materials, mostly carbon nanotube (CNT), or graphene are used as an active material [31,32,33,39,40]. Because CNT and graphene surfaces can be easily tuned by using various functional groups to produce variety wearable sensors. Polymer containing conjugated group which can easily conjugate with CNT and graphene such as pyrene can be coated on CNT and graphene surface to demonstrate various sensors such as ion, polymer, and gas sensors.

Based on these various materials, wearable sensors have been rapidly developed. Table 1 summarizes materials and properties of wearable sensors which were introduced in this review.

These smart sensors monitor health conditions in real time, and the diagnostic results are transmitted to control and processing units. To accomplish this, both wired and wireless systems are used extensively to transmit health-related data for subsequent diagnosis. Wireless systems are preferred because wired systems impair the user’s mobility and comfort and increase the risk of the occurrence of a transmission failure [32,41]. Another advantage of the wireless systems is that they also can realize the telemedicine to a doctor such as continuous diagnosis and scheduling an appointment.

In this paper, various smart sensors and wireless systems are reviewed, the current state of research related to such systems is reported, and their detection mechanisms are compared. Our focus was limited to wearable and attachable sensors, so we did not address implantable sensors, such as those implanted in the skin, tissue, or brain. Section 1 presents the various smart sensors divided into two major categories, i.e., physical sensors for detecting temperature, pressure, and strain and chemical sensors for detecting gases, ions, and biomolecules. In Section 2, we describe multiplexed sensors that can monitor several physiological signals simultaneously. Section 3 provides a discussion about short-range wireless systems including bluetooth, near field communication (NFC), and resonance antenna systems for wearable electronic devices. Our conclusions are presented in Section 4.

## 2. Individual Sensors

### 2.1. Physical Sensors

Physical sensors are capable of sensing physical factors, such as temperature, pressure, and strain. Recently, wearable physical sensors have been developed that have sufficient flexibility and stretchability to resist being deformed by human activities [3,42,43]. The physical factors measured by wearable sensors indicate a person’s condition, including body and skin temperature, blood pressure, pulse rate, and skin strain. All of these factors are important indicators of a person’s health status. Since physical sensors can measure these types of physical factors, they can be used for electronic monitoring of the person’s skin, the human-machine interface, the person’s activities, and personal healthcare applications [3,6,39,44,45].

#### 2.1.1. Temperature Sensors

Body temperature is one of the most fundamental factors associated with human health and physical activity. For example, changes in the temperature can be indicative of pathological symptoms, such as infection, inflammation, hyperthermia, and hypothermia. In addition, real-time monitoring of body temperature is crucial for recognizing sudden adverse occurrences, such as heart attacks. Furthermore, temperature monitoring is important for a soldier and an athlete, where physical activity is directly concerned with their accomplishment.

As they are closely related to the environment and human life, temperature sensors have been studied extensively in various applications. Several methods can be used to fabricate temperature sensors, and they can be divided into several different types, such as pyroelectric temperature detectors, resistive temperature detectors, and thermistors. In addition to the studies related to temperature sensors, other related research has been conducted. One example is the development of thermocouples based on the principle of the Seebeck effect [46] in which a thermal detector that uses thermochromic materials that change colors when the temperature changes [47,48]. In order to utilize these temperature sensors for monitoring people’s health and activities, they must be flexible and stretchable so that they can be attached successfully on the person to be monitored. In recent years, flexibility and stretchability have been achieved by using polymers as the active material and by reconfiguring the structure. The advances in the mechanical properties of these devices have made wearable temperature sensors a reality.

Pyroelectric temperature detectors are fabricated to take advantage of the pyroelectric effect, which is the generation of an electric field by the spontaneous polarization of the material due to changes in the temperature. Lithium tantalate (LiTaO_3_) and barium titanate (BaTiO_3_) are typical pyroelectric materials in which the crystal lattice group of the unit cell has intrinsic polarity. Because these pyroelectric materials are rigid crystals, it is difficult to apply them to flexible, stretchable electronics. To overcome this problem, polymer-based pyroelectric materials, such as polyvinylidene fluoride (PVDF) and its copolymers, have been investigated and used as wearable temperature sensors [49,50,51]. Cosseddu et al. demonstrated a temperature transducer based on an organic thin-film transistor (OTFT) by detecting the pyroelectric effect of PVDF [52]. Because of the pyroelectric properties of the material, a charge separation is produced across the PVDF film when the temperature varies, which is inducing the current variation in the OTFT. Since PVDF and its copolymer, poly(vinylidenefluoride-trifluoroethylene) (P(VDF-TrFE)), have piezoelectric properties as well as pyroelectric properties, they can detect both pressure and temperature as target sensing parameters. Tien et al. demonstrated a flexible, bimodal sensor array to detect both temperature and pressure simultaneously by using a mixture of P(VDF-TrFE) and BaTiO_3_ nanoparticles as a gate dielectric material [53]. By applying alternating current (AC) gate bias to the field-effect transistor (FET), effective remnant polarization of the piezo-pyroelectric gate dielectric layer has been measured based on the amplitude and offset values of the modulated drain current.

**Figure 1:**
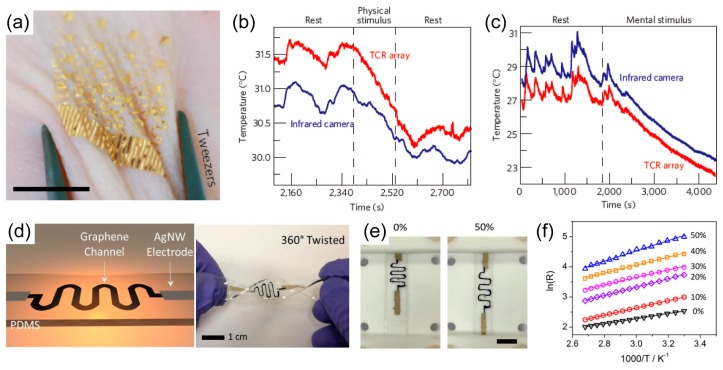
Wearable temperature sensors. (**a**) Image of a 4 × 4 temperature coefficient of resistance (TCR) sensor array after application to the skin deformed by pinching the skin in a twisting motion (scale bar 8 mm). (**b**) Temperature of the palm measured with an infrared camera (blue) and a sensor array (red, offset for clarity) during mental and (**c**) physical stimulus tests. Reprinted with permission from Ref. [42]. Copyright 2016, Nature Publishing Group. (**d**) Schematic diagram and representative image of the stretchable graphene thermistors at twisted states (scale bar 1 cm). (**e**) Images of the stretchable graphene thermistor at 0% and 50% strains (scale bar 1 cm). (**f**) Resistance variation with temperature (30 to 100 °C) within 0% to 50% strains (step 10%). Reprinted with permission from Ref. [31]. Copyright 2015, American Chemical Society.

Resistive temperature detectors (RTDs) are one of the most widely used types of temperature sensors. Typically, a pure metal, usually platinum or copper, is used as the resistive material. The resistance of the metal increases linearly as the temperature increases according to the temperature coefficient of resistance [42,54,55,56,57,58,59]; therefore, the metal-based resistive temperature sensors can detect the changes in the temperature according to the following equation, *R* = *R*_0_[1 + *α*(*T* − *T*_0_)], where *R* is resistance of the sensor at temperature “*T*”, *R*_0_ is resistance of the sensor at reference temperature, usually 20 °C, *α* is the temperature coefficient of resistance, *T* is the temperature of the sensor, and *T*_0_ is reference temperature. Kim et al. demonstrated surgical suture strips that had platinum temperature sensors with Si diodes for a diagnostic system [54]. Serpentine-structured platinum has a linear resistance change of 2.7 Ω/°C as the temperature changes. In particular, an in vivo animal experiment was performed to demonstrate scalability and biocompatibility, and four temperature sensors in the sutures demonstrated stable measurements of the local temperature. Webb et al. fabricated ultrathin conformal devices with gold serpentine-structured temperature sensor arrays (Figure 1a) [42]. Four-by-four arrays of the temperature coefficient of resistance (TCR) can be used as both temperature sensors and local microscale heaters since the electrode can be Joule heated. Subtle variations of the temperature of human skin, which can occur due to physical stimuli, mental activity, vasoconstriction, and dilation, can be determined by TCR array devices in real time (Figure 1b,c).

However, metal-based RTDs have low temperature resolution for wearable applications, which are used to monitor temperatures in the range of 30–40 °C. Unlike metal-based RTDs, the resistance of thermistor-type temperature sensors changes nonlinearly according to the temperature according to the following equation, *R* = *R*_0_ exp(*B*/*T*), where *R* is the resistance at temperature *T*, *R*_0_ is the resistance at *T* = ∞, *B* is the thermal index, *T* is the temperature of the sensor. This feature makes it possible for these sensors to measure temperature much more precisely than RTDs, but the temperature range is limited. The resistance of thermistor-type temperature sensors changes nonlinearly according to the temperature, which makes it possible for these sensors to measure temperature much more precisely than RTDs, but the temperature range is limited. In addition, they have a significant advantage in bio-applications because they have high sensitivity and low cost [31,60,61,62,63,64]. Yu et al. fabricated a stretchable temperature sensor by transferring the elastically-buckled, thin-film thermistors to elastomeric materials [61]. The thermistors were fabricated on silicon-on-insulator and transferred to a pre-strained polydimethylsiloxane (PDMS) substrate to form a buckled structure, and they had negligible resistance changes under the 30% of strain. Yan et al. demonstrated a stretchable graphene thermistor with a graphene thermal channel and silver nanowires (AgNWs) electrode (Figure 1d–f) [31]. The graphene thermal channel shows negative temperature coefficient like a semiconductor. The graphene thermistor embedded in the PDMS not only can be stretched up to 50% but also can be 360° twisted, where the twist is one of the most important properties of wearable electronic devices. Although the offset resistance of the graphene thermal channel changes depending on the applied strain up to 50%, it can be modified by tuning the thermal index.

#### 2.1.2. Pressure Sensors

The pressure ranges are very different depending on the body part or purpose. The pressure ranges of the wearable pressure sensors are divided into three categories, i.e., low-pressure range (<10 kPa), medium-pressure range (10 to 100 kPa), and high-pressure range (>100 kPa) [65]. The low-pressure range (<10 kPa), which corresponds to both the intraocular and intracranial pressures, is an important range because it encompasses the intra-body pressure [6,32]. The type of body pressure that corresponds to the medium-pressure range (10 to 100 kPa) includes blood pressure, heart rate, the radial artery wave, phonation vibration, and skin modulus [66,67,68]. The high-pressure range (>100 kPa) includes the weight of a person or atmospheric pressure at high altitudes [69]. By measuring these various types of pressure, we can monitor eye disease, heart disease, damaged vocal cords, and exercise. Thus, wearable pressure sensors have been studied extensively for applications in healthcare and medical diagnosis devices. Various sensing mechanisms, including piezoelectric, piezoresistive, and capacitance mechanisms turn physical stimuli into electrical signals.

**Figure 2:**
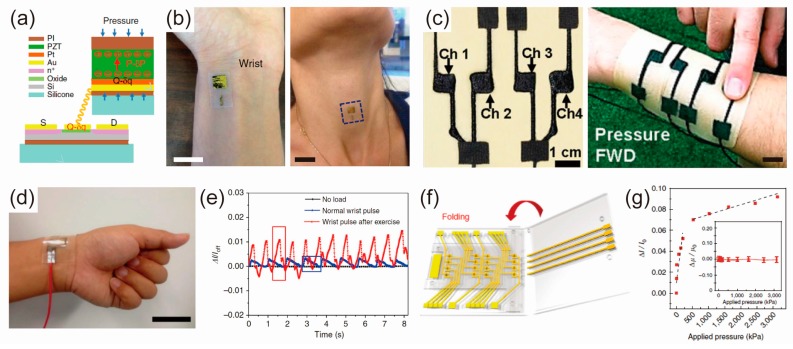
Wearable pressure sensors. (**a**) Cross-sectional schematic illustration of the pressure sensor and its connections to an associated transistor. (**b**) Photograph of the pressure sensor placed on a wrist and neck for measuring fast transients in the blood pressure (scale bars 1 cm and 2 cm). Reprinted with permission from Ref. [3]. Copyright 2014, Nature Publishing Group. (**c**) Images of pressure sensor printed on the commercial elastomeric patch. The sensor array is composed of four channels of pressure sensors (scale bars 1 cm). Reprinted with permission from Ref. [39]. Copyright 2014, John Wiley and Sons. (**d**) Photograph showing the skin-attachable sensor directly above the artery of the wrist (scale bar 3 cm). (**e**) Measurement of the physical force of a heartbeat under normal and exercise conditions. Reprinted with permission from Ref. [36]. Copyright 2014, Nature Publishing Group. (**f**) Schematic image of pressure-sensitive graphene FETs with air-dielectric layers. (**g**) Plot of normalized drain current changes versus applied pressure. (inset indicates relative change in the field effect mobility under applied pressure). Reprinted with permission from Ref. [33]. Copyright 2017, Nature Publishing Group.

The principle of piezoelectric-based pressure is based on piezoelectric effects that are electrical charges that occur in certain types of solid materials under pressure. The electricity induced by pressure can be expressed by the following equations: *D_i_* = *ε*_0_*ε_ij_^σ^E_i_* + *d_iJ_σ_J_*, and *δ_I_* = *S_IJ_^E^σ_J_* + *d_Ii_E_i_*. *D*, *E*, *σ*, *δ*, *ε*_0_, *d*, *ε^σ^*, and *S^E^* represent the electric displacement, the electric field, the stress by pressure, the strain by pressure, the free-space electric permittivity, the piezoelectric coefficient, the electric relative permittivity, and the mechanical compliance matrix. *I*, *J*, *i*, and *j* are indices. The *d*_31_ and *d*_33_ is known as the transverse and longitudinal coefficient, respectively. Therefore, the piezoelectric effect means that the dipole forms a polarization when pressure is applied to the materials, and this polarization is proportional to the applied pressure. Due to their fast response time and low power consumption, piezoelectric pressure sensors are used extensively in the detection of dynamic pressures, such as the vibrations of sound. Typical materials used for piezoelectric pressure sensors are lead titanate (PbTiO_3_), BaTiO3, PVDF and P(VDF-TrFE). Particularly, a film type sensor is used extensively for manufacturing a wearable pressure sensor, and a representative material is P(VDF-TrFE) [51,70,71,72]. Persano et al. introduced a large-area, flexible pressure sensor through the free-standing aligned array of P(VDF-TrFE) nanofibers [72]. The sensor demonstrated ultra-high sensitivity to pressure changes, even very small changes, e.g., 0.1 Pa. Recently, a pressure sensor was combined with a transistor to improve the performances of the devices [3,67]. Dagdeviren et al. reported a stretchable, ultrathin pressure sensor based on PbZr_0.52_Ti_0.48_O_3_ (PZT) that conforms nicely to the skin. The ultrathin and high-quality PZT sheet is used as a component of a capacitor that was connected to the gate electrodes of a FET (Figure 2a) [3]. FETs amplify the piezoelectric response of the PZT and convert it to an output current. The device is very thin (25 μm) and lightweight (2 mg), and it has high sensitivity (~0.005 Pa) and fast response time (0.1 ms). For monitoring blood pressure, the device can be softly laminated on the wrist, neck, or throat (Figure 2b).

The piezo-resistive pressure sensor is based on the fact that the resistance of the material changes with the applied pressure. The resistance change of the material can be expressed by the equation Δ*R*/*R* = (1 + 2*ν*)*ε* + (Δ*ρ*/*ρ*), where (1 + 2*ν*), *ε*, and (Δ*ρ*/*ρ*) are geometric effect term, strain by pressure, and resistivity effect term, respectively. That is, the change in resistance originates from a change in dimension by pressure. The pressure sensor using this mechanism is very simple to manufacture, and it has been studied extensively because it can detect a wide range of pressures. Usually, conductive fillers (reduced graphene oxides (rGO), carbon nanotubes (CNTs), or metal particles) are added to the elastomer to produce materials with piezoresistive properties, but these sensors have low sensitivity. To solve this problem, studies have been conducted to increase the sensitivity using a microstructure or a porous structure. Park et al. introduced stretchable pressure sensors using the unique geometry of interlocked microdome structures [45,73]. These pressure sensors can be attached to the skin and distinguish a variety of mechanical stimuli. However, the fabrication method requires the use of an Si mold, which is complicated, expensive, and has poor reproducibility. Therefore, Jung et al. developed a wearable piezoresistive pressure sensor by creating pores in a conventional pressure-sensitive rubber (PSR) [39]. Sensors based on these methods have greater sensitivity than conventional PSR-based sensors. This wearable porous PSR pressure sensor successfully conforms to the skin for various applications, such as human-machine interfaces, healthcare monitoring, and radio control of robots (Figure 2c). In addition to the aforementioned pressure sensors based on the microstructure and porous structures, piezoresistive pressure sensors based on other materials also have been developed [36,74]. Gong et al. reported a flexible pressure sensor with high sensitivity by interposing the gold nanowires (AuNWs) between two PDMS films [36]. The AuNW-based pressure sensors provide real-time monitoring of blood pressure with high sensitivity and flexibility, as shown in Figure 2d,e.

Capacitive pressure sensors use capacitors, and their capacitance values vary with the thickness of dielectric materials. The capacitance *C* is given by the equation *C* = *ε*_0_*ε_r_*(*A*/*d*), where *ε*_0_ is the electric constant, *ε_r_* is the relative static permittivity of the dielectric, *A* is the area of the overlap of the two plates, and d is the thickness of the dielectric. When the thickness is reduced by pressure, the capacitance increases. In order to change the thickness depending on the pressure, materials with small modulus values, such as PDMS, ecoflex, and polyurethane (PU), usually are used. Sun et al. developed a pressure sensor that includes a transparent, stretchable dielectric that is sandwiched between two flexible ion conductors [75]. This sensor can be used for wearable or implantable electronic applications due its transparency, stretchability, and biocompatibility. In addition, Park et al. developed a flexible, capacitive pressure sensor with very high sensitivity by constructing an air gap between a porous PDMS film and a single-wall carbon nanotube (SWCNT) film [76]. However, capacitive-type pressure sensors have limitations, such as low sensitivity and slow response time, because of the small modulus of elastomer. The sensitivity can be improved through the use of an air dielectric layer. Therefore, Zang et al. demonstrated a flexible pressure sensor based on the suspended gate electrode of the FET [77]. The sensor has very high sensitivity to low pressures and can be attached to the wrist to monitor pulse waves. The abovementioned pressure sensors are sensitive and can detect specific pressure ranges. However, there is a need for a pressure sensor that can sense a wide range of pressures for a variety of applications. Recently, Shin et al. reported an unconventional approach to fabricating a pressure sensor array with an air dielectric layer formed by folding panels (Figure 2f) [33]. The sensor can be used for a wide range of tactile sensing, as shown in Figure 2g. These results demonstrated that pressure sensing devices are the essential element for monitoring human activity and personal healthcare.

#### 2.1.3. Strain Sensors

Small and large deformations can occur when the person is moving. In wearable electronics, the strain sensor has the significant role of monitoring the motion of the person’s body, ranging from the vibration of the vocal cords to the movements of joints. Thus, strain sensors have been designed and developed to be flexible, stretchable materials, quite different from the previously-studied, silicon-based strain sensors [78]. The requirements for wearable strain sensors include stretchability, sensitivity (gauge factor), mechanical reliability, hysteresis, and a linear output signal [79]. Wearable strain sensors can be specified according to the principles that use for sensing, e.g., there are piezoresistive, capacitive, and piezoelectric strain sensors.

**Figure 3:**
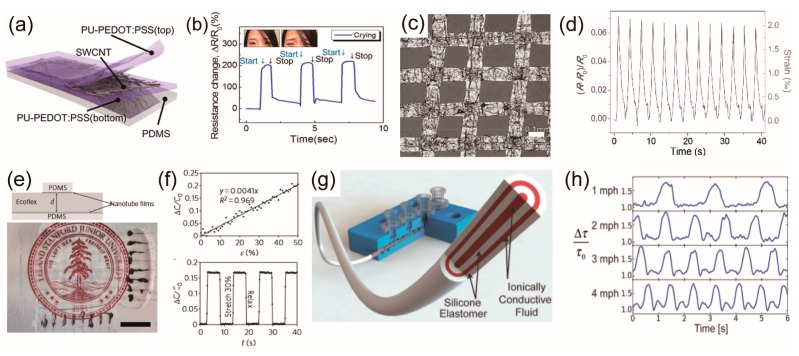
Wearable strain sensors. (**a**) Schematic illustration of the cross-section of the strain sensor consisting of the three-layer stacked nano hybrid structure of polyurethane-poly(3,4-ethylenedioxythiophene) polystyrenesulfonate (PU-PEDOT:PSS)/single-wall carbon nanotube (SWCNT)/PU-PEDOT:PSS on a polydimethylsiloxane (PDMS) substrate. (**b**) Time-dependent Δ*R*/*R*_0_ responses of the sensor attached to the forehead when the subject was crying. Reprinted with permission from Ref. [34]. Copyright 2015, American Chemical Society. (**c**) Optical micrograph of a graphene woven fabrics (GWFs)-PDMS-tape composite film (scale bar 0.1 mm). (**d**) Relative change of resistance between 0% and 0.2% strain. Reprinted with permission from Ref. [86]. Copyright 2014, John Wiley and Sons. (**e**) Schematic illustration of stretchable capacitor with transparent electrode (top) and photograph of the same device reversibly adhered to a backlit liquid-crystal display (bottom) (scale bar 1 cm). (**f**) Change in capacitance Δ*C*/*C*_0_ versus strain ε (top) and Δ*C*/*C*_0_ versus time t over four cycles of stretching (bottom). Reprinted with permission from Ref. [69]. Copyright 2011, Nature Publishing Group. (**g**) Schematic image of multicore-shell printing process for fiber-type capacitive strain sensor. (**h**) Normalized decay time output of the sensor for different walking speeds up to 4 mph. Reprinted with permission from Ref. [92]. Copyright 2015, John Wiley and Sons.

Piezoresistive strain sensors detect deformations mainly by changes in the resistivity. The factors associated with the change in the resistance of materials used in piezoresistive strain sensors can be explained by the simple equation, *R* = *ρ*(*l*/*A*), where *ρ* is resistivity, *l* is length, and *A* is the cross-sectional area of the material. Both *l* and *A* are geometrical factors, and changes in ρ are induced by the piezoresistivity of the materials themselves. During the deformation of metals and semiconductors, the bandgap changes because of the change in the interatomic spacing [80]. Consequently, Δ*R*/*R* becomes gauge factor of strain sensors. Semiconductors have a high gauge factor due to their large changes in resistivity, so many semiconducting materials are used for piezoresistive strain sensing materials, such as zinc oxide (ZnO), carbon black, and CNT [34,81,82,83]. Roh et al. fabricated strain sensors by embedding single-wall carbon nanotubes in a conductive elastomer polyurethane-poly(3,4-ethylenedioxythiophene) polystyrenesulfonate (PU-PEDOT:PSS), as shown in Figure 3a. This sensor has the stretchability of 100% strain, 62% transmittance in the visible range, and a gauge factor of 62. (Figure 3b) [34]. In addition, changes in the resistance are possible due to disconnections in the network geometry or cracks that may occur during deformation. In the case of conductive nanomaterial networks, such as metal nanowires (mNWs), graphene flakes or films, and CNTs, sliding can occur within the network during stretching or bending, and it results in significant changes in the resistance [84,85,86,87,88,89]. For example, graphene woven fabric (GWF) was made and used as a strain sensor (Figure 3c,d), and the sensor had gauge factors of 10^3^ at strains of 2–6% and 10^6^ at higher strains (>7%) because of the high-density cracks that were generated in the network during stretching [86].

Capacitive strain sensors detect strain based on changes in the capacitance that occur due to the geometrical effect. Capacitive strain sensors usually have two electrode pads separated by an elastomeric dielectric layer. The capacitance of strain sensor can be expressed with the equation *C*_0_ = *ε*_0_*ε_r_l*_0_*w*_0_/*d*_0_, *ε*_0_ and *ε_r_* is the dielectric constants and *l*_0_, *w*_0_, *d*_0_ are length, width, thickness of the active area of the sensor, respectively. When the sensor is stretched with the strain *ε*, the length becomes *l* = (1 + *ε*)*l*_0_ but the width and thickness decrease up to the poisson’s ratio *ν*, *w* = (1 − *ν*)*w*_0_ and *d* = (1 − *ν*)*d*_0_ thus capacitive increases to *C* = (1 + *ε*)*C*_0_. To ensure stretchability in wearable applications, stretchable transparent electrodes usually are used, such as mNWs or CNTs [69,90,91,92,93,94]. Lipomi et al. demonstrated a stretchable and transparent strain sensor using CNTs and silicon elastomer (Figure 3e,f) [69]. Frutiger et al. fabricated a fiber-shaped, capacitive strain sensor for wearable electronics using silicon elastomer and ionic conductive fluid (Figure 3g,h). The gauge factor was 0.348, and the sensor had 700% stretchability [92].

The piezoelectric effect of certain materials has been applied for sensing strain. Piezoelectric strain sensors have been developed using various oxides, such as ZnO and zinc stannate (ZnSnO_3_) or piezoelectric polymers, such as PVDF and P(VDF-TrFE) [95,96,97,98]. Wu et al. demonstrated ZnSnO_3_ nano/microwire-based piezoelectric sensors that were flexible and had a gauge factor of 3740 at 0.35% strain [95]. Piezoelectric strain sensors usually are self-powered, thus they do not require an additional battery or power supply. However, the mechanical properties of piezoelectric materials limit the application of such sensors in wearable electronics, which require stretchability, so there are still challenges to be overcome [98].

### 2.2. Chemical Sensors

Recently, wearable sensors that are capable of detecting of various signals have attracted substantial research interest because of their potential application in healthcare [32,38,99] and environmental monitoring [18,21]. The chemical signals from environmental pollutants, chemical and biological warfare agents, and clinically-relevant biomarkers are related closely to the chemical signals from the human body [100,101,102], and, as a result, there is significant expectation that chemical sensors will eventually have a large share of the market for wearable sensors. Currently, the available commercial medical devices used to extract bodily fluids from patients, such as blood, urine, saliva, or tears cause pain and inconvenience for the patients [103]. Also, 24-h monitoring is required for certain diseases, so the patients’ daily activities are restricted. Thus, research on chemical sensors that can be worn or attached to the body is a high priority in the healthcare field because it offers hope for minimizing the inconvenience of that user’s experience, and it will enable 24-h monitoring of the status of patients.

#### 2.2.1. Gas Sensors

With the increase in the problems associated with environmental pollution and threat of the use of biochemical weapons, it is highly desirable to have sensors that can detect the molecules of various gases, which would enhance our ability to protect people by providing information concerning the presence of hazardous gases at specific places and sounding alarm signals to protect the public [104,105]. To develop such a system, it is necessary to study wearable gas sensors that are light, inexpensive, and effective in providing timely warnings. This could be done by attaching gas sensors to arbitrary surfaces such as human skin, portable devices, live plants, and insects. There are many sensing mechanisms for detecting gas molecules, such as resistive mechanisms [106], piezoelectric devices [107], surface acoustic waves [108], electrochemical processes [109], and colorimetric methods [110]. Among the various sensing methods, wearable resistive, electrochemical, and colorimetric types of gas sensors have been studied extensively.

**Figure 4:**
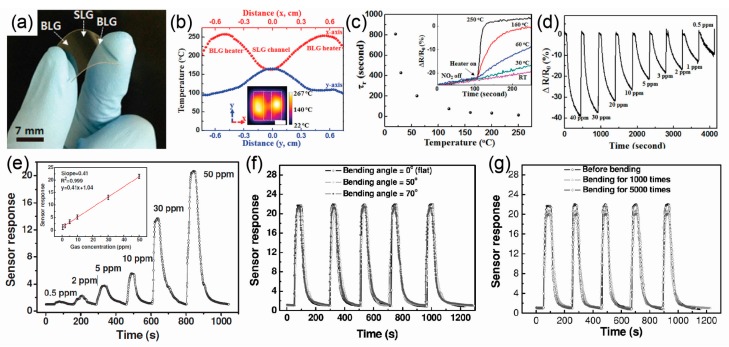

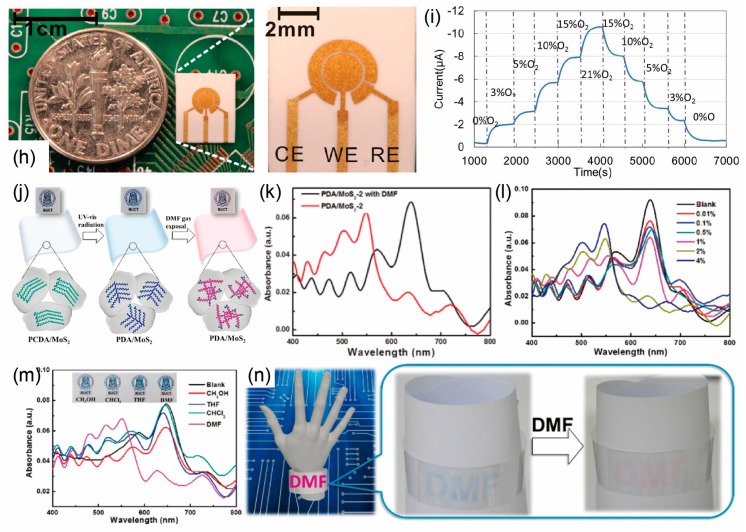
Wearable gas sensors and its integrated systems. (**a**) Photograph of transparent and flexible single-layer graphene (SLG) sensor channel-bilayer graphene (BLG) heater on a polyethersulfone (PES) substrate (scale bar 7 mm). (**b**)Temperature distribution along transverse (x-axis) and longitudinal (y-axis) direction of sensor-heater device structured as laterally intercalated SLG sensor channel (6 mm width) between BLG heaters (7 mm width) with applied 1.7 W of electric power. Here the red dot and blue dot are temperature profiles of thermal image in inset along x-axis and y-axis with origin at center on channel, respectively. Inset: Spatial temperature distribution of graphene heaters (7 mm width) which intercalate 6 mm width graphene sensor with applied 1.7 W. Here three broken squares indicate center channel and side heaters area, respectively (scale bar 7 mm). (**c**) Recovering time constant *τ_r_* as a function of heater temperature. Inset: the recovering curves of the Δ*R*/*R*_0_ as a function of time under different temperature range from room temperature to 250 °C. (**d**) The relative resistance variation Δ*R*/*R*_0_ of SLG channels as a function of time including recovery step with 100 to 165 °C heating under different NO_2_ gas concentration from 40 to 0.5 ppm. Reprinted with permission from Ref. [37] Copyright 2014, John Wiley and Sons. (**e**) Response curves of the sensor to NO_2_ of different concentrations. Inset: The sensor response depends linearly on NO_2_ concentration. (**f**) Response curves of the sensor to 50 ppm of NO_2_ when tested under different bending angles. (**g**) Response curves of the sensor tested before and after bending 1000 and 5000 times (bending angle = 50°). Reprinted with permission from Ref. [114] Copyright 2014, John Wiley and Sons. (**h**) Photograph of microfabricated flexible room temperature ionic liquid (RTIL) based gas sensor (scale bars 1 cm and 2 mm, respectively). (**i**) Current versus time curve at various oxygen concentrations when the potential is held at −1.4 V vs. Au. Nitrogen is the background gas. Oxygen concentration steps up from 0% to 21% and steps down from 21% to 0%. Reprinted with permission from Ref. [117] Copyright 2013, IEEE. (**j**) Schematic illustration of the preparation of the PDA/MoS_2_ film and the sensor upon exposure to DMF vapor. (**k**) UV-vis spectra of polydiacetylene (PDA)/MoS_2_ composites with an increased ratio of MoS_2_ to PDA in the absence and presence of 0.1% DMF vapor. (**l**) UV-vis spectra of PDA/MoS_2_ films exposed to *N*,*N*-dimethylformamide (DMF) vapor with different concentrations. (**m**) UV-vis spectra of the PDA/MoS_2_ film upon exposure to different (5%) vapors, in comparison with 2% DMF vapor. (**n**) Flexible transparent wrist strap with DMF sensing ability. Reprinted with permission from Ref. [110] Copyright 2017, Royal Society of Chemistry.

A resistive-type gas sensor operates based on the difference in the electrical resistance of various materials, such as metal oxides, conducting polymers, and graphene. Recently, graphene has been used extensively in wearable gas sensors due to its outstanding flexibility and high surface-to-volume ratio and its electrical response to adsorbed gas molecules on sp^2^-bonded carbon networks [18,111,112,113]. However, gas phase detection with graphene sensors is generally irreversible because this system relies on the adsorption of gas molecules on the graphene, and complete desorption of the adsorbed gas molecules is very challenging. In an attempt to solve this problem, Choi et al. reported centimetre-scale transparent graphene sensors for nitrogen dioxide (NO_2_) gas that had laterally-integrated or vertically-integrated graphene heaters on a polyethersulfone (PES) substrate [37]. Figure 4a shows a photograph of a transparent, flexible single-layer graphene (SLG) gas sensor with built-in, bilayer graphene (BLG) heaters. Figure 4b shows that the temperature of the SLG sensor can reach 165 °C when the BLG heater temperature is 250 °C. Figure 4c shows the recovery time constant (*τ_r_*) after using the heaters to apply different temperatures. The *τ_r_* can be extracted from the equation as ∆*R*/*R*_0_ (*t*) = ∆*R*/*R*_0_ (*t*_0_) exp[*−*(*t − t*_0_)/*τ*], where ∆*R*/*R*_0_ is the change in the relative resistance, and *t*_0_ is the time required to change the curvature of ∆*R*/*R*_0_. When the BLG heater was operated to increase the temperature (*T*) of the SLG sensor, the *τ_r_* was drastically shorter than that of room temperature (*T* < 100 °C, *τ_r_* > 100 s, but for *T* < 250 °C, *τ_r_* ≈ 11 s). This combined sensor-heater device can detect concentrations of NO_2_ gas in the range of 0.5 to 40 ppm (Figure 4d). In addition to graphene based gas sensors, Lui et al. demonstrated colloidal quantum dots (CQDs) based resistive type gas sensors for NO_2_ gas detection [114]. Because CQDs are nanocrystals of a few nanometers, they have an extremely large surface-to-volume ratio capable of active interaction with target gas molecules [115]. Lead sulfide (PbS) CQDs were spin-coated on the gold electrodes patterned paper substrates for highly flexible gas sensors. Moreover, to improve the gas sensing performances, oleate ligands on the surface of PbS CQD was removed by sodium nitrite (NaNO_2_) treatment, which contributed to enhancing the accessibility of CQD surfaces to gas molecules. Figure 4e shows the dynamic response of the gas sensors upon NO_2_ exposure/removal cycles with NO_2_ concentration of 0.5, 2, 5, 10, 30, and 50 ppm, respectively. As shown in the inset of Figure 4e, the sensor exhibited a sensitivity of 0.41 ppm^−1^. Figure 4f,g show the bending and fatigue properties of gas sensors. As shown in Figure 4f, the response and reversibility of the sensor were fully retained at the bending angles of 50° and 70°. Also, the gas sensor exhibits excellent reliability under repeated bending cycles of 5000 (bending angles of 50°).

An electrochemical gas sensor operates by reacting with gas molecules and creating an electrical signal. This sensor consists of a sensing electrode and a counter electrode that are separated by an electrolyte. When the target gas diffuses into the sensors, through the porous membrane to the working electrodes where it is oxidized or reduced. This electrochemical reaction generates an electric current flow between the sensing and counter electrodes. Electrochemical gas sensors are among the most promising approaches for wearable devices due to their low power consumption, low cost, high sensitivity, and high selectivity. Also, they can be operated easily by using a single microelectronics chip [116]. However, conventional electrochemical sensors have limited lifetimes because they use volatile electrolytes. To overcome this limitation, many researchers have replaced the volatile electrolytes with room temperature ionic liquids (RTILs). Unlike conventional electrolytes, RTILs are non-volatile, and they have negligible vapor pressure and high thermal stability. Mu et al. demonstrated a flexible electrochemical oxygen (O_2_) gas sensor using an RTIL as the electrolyte with a porous polytetrafluoroethylene (PTFE) substrate [117]. The porous structure of the PTFE substrate is suitable for an electrochemical gas sensor membrane due to its outstanding chemical resistance, flexibility, and gas permeability. Figure 4e shows a micro-fabricated, flexible, RTIL-based gas sensor. An O_2_ gas sensor can respond linearly to O_2_ gas concentrations ranging from 0 to 21%, and Figure 4f shows that the sensor recovered fully from these responses when the O_2_ gas was removed.

In addition to the sensing mechanism mentioned above, colorimetric gas sensors are capable of producing a visually-recognizable signal, and they do not have some of the disadvantages associated with conventional gas sensors due to electrical issues with power sources, displays, and electronic circuits. Recently, Wang et al. reported a flexible, transparent, colorimetric *N*,*N*-dimethylformamide (DMF) gas sensor using a hierarchical polydiacetylene/molybdenum disulfide (PDA/MoS_2_) nanocomposite film [110]. PDA, as a typical conjugated polymer for colorimetric sensors, has novel properties of color and changes in fluorescence in response to external stimuli, e.g., temperature, pH, and chemical stress. MoS_2_, with its nanoflake form, acts as a supporter for PDA films, thereby enhancing the surface-to-volume ratio. Figure 4g shows the fabrication process of PDA/MoS_2_ composite film and the sensor upon exposure to DMF gas. When the PDA/MoS_2_ composite was exposed to 0.1% DMF vapor, the peak intensity of optical adsorption at ~640 nm (blue) decreased, and another peak appeared at ~550 nm (red) (Figure 4h). A quantitative measure for the blue to red transition was given by the colorimetric response (CR), which was defined as *B_i_* = *A*_blue_/(*A*_blue_ + *A*_red_), where *A*_blue_ was the absorbance at ~640 nm and *A*_red_ was the absorbance at ~550 nm. The *B_i_* of the PDA/MoS_2_ composite film in the absence and presence of an DMF gas was recorded to calculate the vapor-induced CR. CR = (*B*_0_ − *B*_1_)/*B*_0_ × 100%, where *B*_0_ and *B*_1_ were the percent of blue before and after DMF gas exposure, respectively. As the concentration of the DMF vapor increased, the absorption peak at ~640 nm gradually was weakened, and this was accompanied by increased intensity of the adsorption peak at ~550 nm, indicating that the PDA/MoS_2_ has outstanding sensitivity to DMF vapor in the concentration range of 0.01% to 4% (Figure 4i). Figure 4j shows the performance of sensors upon exposure to various vapors, including of methanol, chloroform, tetrahydrofuran, and DMF. The colour of the PDA/MoS_2_ composite film changed to red in DMF vapor, but no changes in colour were observed in other vapors. As shown in Figure 4k, wearable wrist-strap DMF gas sensors based on the PDA/MoS_2_ composite were demonstrated with high transparency, vivid colour changes, and outstanding flexibility and reliability, all of which suggests this composite has great potential for smart wearable devices. Although colorimetric gas sensors have provided a promising strategy for producing wearable gas sensors, there are still substantial challenges to their practical application, such as limits on the quantitative display of gas concentrations and the recovery of the sensors after removing target gases.

#### 2.2.2. Ion Sensors

In recent years, personalized healthcare systems have been emphasized substantially and explored extensively by many researchers, targeting people who desire to monitor their physiological status continuously and wish to detect any pathologies as early as possible. Electrolyte imbalances can be indicative of potential health problems, such as hyperkalemia, cystic fibrosis, physical stress, osteoporosis, and mineral loss in bones [118]. Thus, monitoring the core electrolytes, e.g., H^+^, Na^+^, K^+^, Na^+^, Ca^2+^, Fe^3+^, and NH_4_^+^, in biological fluids is very important in the diagnosis of latent diseases. Therefore, a wide variety of wearable devices have been developed to date, including skin patches, wrist watches, and chest bands [119,120].

The most common analytes for wearable electrochemical sensors are pH and ions occupying 33% of whole wearable electrochemical sensors [121]. In addition, healthcare and medical applications account for 46% of the total market. As a result, the development of a wearable device for sensing ions is essential in meeting current social needs. Electrochemical sensors generally utilize one of three representative analytical methods, i.e., potentiometric, amperometric, and voltammetric sensors. Each type of wearable electrochemical ion sensor, classified by the analytical method it uses, is discussed below.

**Figure 5:**
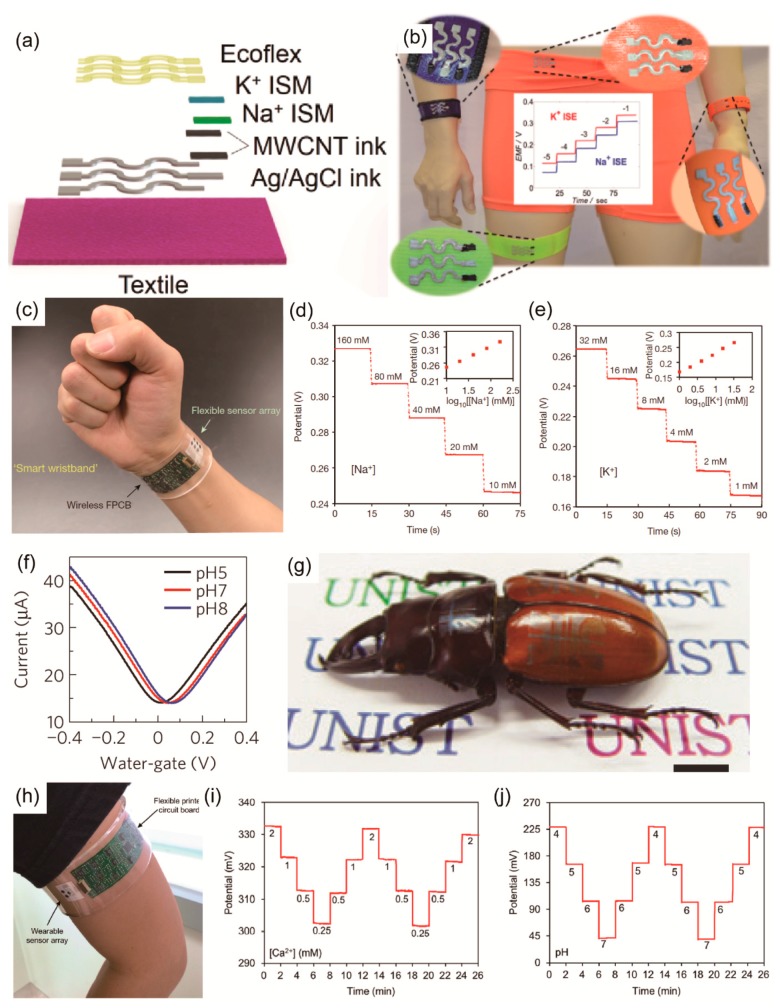
Wearable electrochemical ion sensors and its integrated systems. (**a**) Schematic representation of the tailor-made stretchable materials and manufacturing process. (**b**) Photograph of the printed sensors on different common textiles and typical time trace plots for potassium and sodium. Reprinted with permission from Ref. [122]. Copyright 2016, John Wiley and Sons. (**c**) Photograph of a wearable flexible integrated sensing array and the open circuit potential responses of Na^+^ (**d**) and K^+^ (**e**) sensor, respectively. Reprinted with permission from Ref. [38]. Copyright 2016, Nature Publishing Group. (**f**) Water-gate characterization at different pH levels. (**g**) Photograph of monolithic device structures transferred onto the epidermis of an insect. (scale bar 5 mm). Reprinted with permission from Ref. [126]. Copyright 2012, Nature Publishing Group. (**h**) A fully integrated wearable sensing system on a subject’s arm and general performance of Ca^2+^ (**i**) and pH (**j**) sensors. Reprinted with permission from Ref. [127]. Copyright 2016, American Chemical Society.

Potentiometric sensors are the most used among the three types of sensors, and this is due to their simplicity, people’s familiarity with them, and their comparatively low cost. One electrode is the working electrode, and its potential is determined by its environment. The second electrode is a reference electrode, and its potential is fixed by a solution that contains the ion of interest. Since the reference electrode has a constant potential, the value of the potential difference (cell potential) can be related to the concentration of the dissolved ions. Parrilla et al. fabricated highly-stretchable, textile-based potentiometric multi-ion (K^+^ and Na^+^) sensors using polyurethane (PU)-based ion-selective membranes and inks with a serpentine sensor pattern [122] (Figure 5a). Instead of the common polyvinyl chloride (PVC) matrix, they used a PU membrane to achieve excellent biocompatibility [123] and additional resistance to mechanical stress, while providing exceptional analytical performance when the potentiometric technique was used [124]. For selective penetration of target ions, they fabricated sodium-selective membrane (Na^+^SM) and potassium-selective membrane (K^+^SM). The membranes were prepared by dissolving the mixture in THF (1 mL). Figure 5b shows that the results from both the K^+^ and Na^+^ sensor arrays exhibited dynamic ranges that fully covered the physiological sodium and potassium levels in the subjects’ sweat before, during, and after prolonged exercise. All of the sensors performed favorably within the linear range from 10^−3^ to 10^−1^ M, yielding a near-Nernstian response of the sensor. In addition, for sensing sweat, the sensor can be printed on various textile products that are commonly worn, such armbands, bandages, and underwear. In experiments to determine the resilience of the sensors, excellent mechanical stability was observed when the sensors were stretched, bent, crumpled, and washed in a beaker of water with vigorous agitation (1250 rpm). Overall, the wearable textile potentiometric array provides an attractive analytical performance before and after exposures to severe mechanical stress. As another application of the potentiometric ion sensor, Gao et al. presented wearable, flexible, integrated sensing arrays for simultaneous and selective screening of sodium ions and potassium ions, as well as other sweat metabolites and body temperature [38]. The measurement of Na^+^ and K^+^ levels is facilitated by the use of ion-selective electrodes (ISEs), coupled with a polyvinyl butyral (PVB)-coated reference electrode maintaining a stable potential in solutions with different ionic strengths. Figure 5c shows the integrated wearable sensor system, which is smart wristwatch for real-time monitoring of perspiration. Also, Figure 5d,e indicate that the Na^+^ and K^+^ sensors had almost the same sensitivities, i.e., 62.5 and 59.5 mV per decade of concentration, respectively, in ambient conditions.

Amperometric measurements are made by recording the current flow in the cell at a given applied potential. However, voltammetric measurements exploit the potential difference across an electrochemical cell. When the potential is swept from one set value to another, the current in the cell is plotted as a function of the applied potential. In both cases, the electron transfer is the essential operational mechanism of the devices. Kwan et al. constructed an amperometric ion sensor for the analysis of phosphates in human saliva by immobilizing pyruvate oxidase on a screen-printed electrode [125]. Hydrogen peroxide (H_2_O_2_) is generated by the enzyme reaction, depending on the concentration of phosphate in the human saliva. The sensor responds within 2 s after the addition of a sample of a standard solution sample, and it has a short recovery time (2 min). This means that the time the sensor requires for each measurement is much shorter than the time required by a commercial kit for testing for phosphates, i.e., 2 and 10 min, respectively. As an example of voltammetric methods, Park et al. presented a flexible and semi-transparent monolithic graphene-graphite field-effect transistor (FET) array for real-time sensing of pH [126]. The FET arrays we fabricated were composed of four blocks, each of which had nine individual FETs. The real-time, multiplexed, pH-sensing outputs from each of the nine FET sensors describe complementary sensing mechanisms caused by the change in conductance, which depended on the concentration of the solution, and the sensors exhibited inverted responses in the p-type regime (water-gate voltage, *V_WG_* = −0.1 V) and in the n-type regime (*V_WG_* = 0.3 V). These inverted responses in each region expand the possibility for reducing electrical cross-talk or false-positive signals. While sweeping the silver (Ag)/silver chloride (AgCl) water-gate voltage at different pH levels, the point of charge neutrality shifted positively with increasing pH, and its sensitivity was ~17 mV/pH (Figure 5f). Also, this flexible monolithic device can be integrated with various nonplanar surfaces, such as cylindrical glass tubes, contact lens, glove fingers, coins, and the epidermis of an insect, providing the versatility of flexible, wearable electronics, as shown in Figure 5g.

For another application of voltammetric measurements, Nyein et al. demonstrated a wearable electrochemical sensor for the continuous monitoring of ionized calcium and the pH of body fluids [127]. Measurement of calcium ions is known to be difficult because of the strict experimental procedure and its dependence on the pH value. Actually, ionized calcium binds to negatively charged sites on protein molecules, competing with hydrogen ions for the same binding sites [128]. This mechanism is pH-dependent, and it alters the level of ionized calcium in the blood. Therefore, Nyein et al. fabricated a wearable, electrochemical-sensing platform to detect in a non-invasive and continuous manner the actual Ca^2+^ concentration in body fluids and the pH of these fluids, such as sweat, urine, and tears. Figure 5h shows a fully-integrated, wearable, multiplexed sensing system that is composed of flexible sensor arrays and a flexible printed circuit board. The device is attached directly to human skin so that immediate analysis after the secretion of fluids is possible, and the lack of a time delay minimizes cross-contamination. In this study, the Ca^2+^ sensing electrode consists of a thin organic membrane containing electrically neutral carrier calcium ionophore II (ETH 129) and an ion-electron transducer (PEDOT:PSS), and the pH sensing electrode detects H^+^ by deprotonation at the surface of polyaniline (PANI). The general performances of both Ca^2+^-based and polyaniline (PANI)-based pH sensors were evaluated by in-situ and ex-situ analyses. The sensitivity of an electrochemical Ca^2+^ sensor shows a near-Nernstian response with an average of 32.7 mV/decade, and the sensitivity of the pH sensor exhibited an average slope of 62.5 mV/decade with good selectivity (Figure 5i,j).

#### 2.2.3. Biosensors

Biosensors are analytical devices that are used to detect biological analytes, such as glucose, lactate, DNA, and antibodies, by using physiochemical measurements. Since biological analytes reflect health problems, biosensors are being used in treating patients and providing healthcare. In addition, by making biosensors wearable, they can perform continuous and non-invasive monitoring of the biological analytes. Biosensors use various sensing methods, and wearable biosensors are being developed that use the electrochemical method, the FET-based electrochemical method, and the optical method [129,130].

Electrochemical biosensors are conventional devices that are used to determine the concentration of biological analytes by directly converting a biological event into an electronic signal. These sensors consist of electrodes, which are the electrochemical transducers, and biorecognition materials. The interaction between the biorecognition materials on the electrodes of the sensor and the biological analyte produce changes in the values of the current and the potential, and these changes are measured [131,132,133]. Many fabrication methods have been studied for applying these sensors to wearable devices [119,134,135,136]. Windmiller et al. developed an electrochemical biosensor consisting of electrodes that were transferred onto non-planar surfaces by using stamp [135]. The screen printing approaches, which were published before their work, largely were confined to flat surfaces, which may restrict the potential utility of the method in the fields of electrochemical sensors and biosensors [133,137]. However, they have been used to transfer various conducting and insulating inks used as electrode materials to irregular surfaces, such as human skin, by using an elastomeric stamp that contained the electrode pattern. The biosensor formed on the skin can detect the concentration of uric acid.

**Figure 6:**
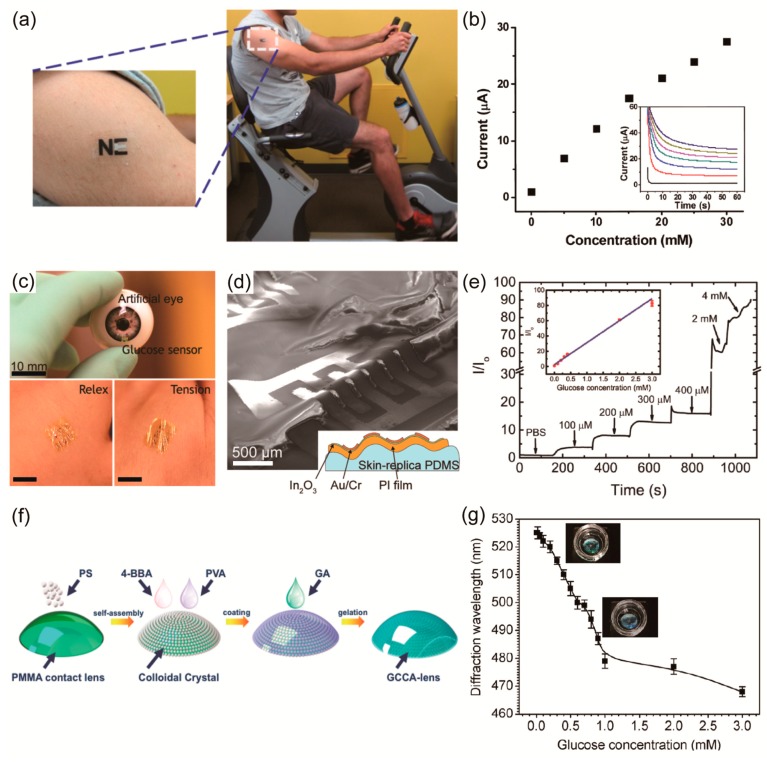
Wearable biosensors (**a**) The images of the temporary transfer-tattoo biosensor attached on deltoid. (**b**) Amperometric response as a function of lactate concentration for the sensor at 37 °C. Inset: profiles of current at different lactate concentrations. Reprinted with permission from [136]. Copyright 2013, American Chemical Society. (**c**) Conceptual images of conformally contacted devices on an artificial eye for glucose sensing in tears are shown. Thin-film sensors remained in contact with skin even during tension and relaxation (scale bars 10 mm). (**d**) Scanning electron microscope image of a representative device (thickness of 1.7 μm) on an artificial PDMS skin replica indicating conformal contact between the device and the substrate (scale bar 500 μm). (**e**) Representative responses of In2O3 sensors to physiologically relevant d-glucose concentrations found in human diabetic tears (lower range) and blood (upper range). Inset: data from five devices. Error bars represent standard deviations of the means. Reprinted with permission from Ref. [40]. Copyright 2013, American Chemical Society. (**f**) The preparation route of the 4-boronobenzaldehyde (4-BBA)-modified poly(vinyl alcohol) (PVA) gelated colloidal crystal array (GCCA)-lens. (**g**) The diffraction response at low glucose concentration. Insert: the photograph of the GCCA-lens sample. Reprinted with permission from Ref. [144]. Copyright 2017, MDPI AG.

Biosensors that can be attached to the skin in the form of tattoos also have been developed [119]. Jia et al. developed a flexible, printed, temporary-transfer tattoo electrochemical biosensor that conforms to the wearer’s skin [136]. They described the first example of real-time, non-invasive, sensing of lactate in human sweat during exercise (Figure 6a). Such direct epidermal monitoring of lactate was conducted using tattoo sensors functionalized with lactate oxidase. Figure 6b shows the resulting calibration curve that indicates the correlation between the current that is measured during evaluation of the epidermis and the absolute lactate concentration. This sensitive sensor exhibited a low noise level even at the 1 mM concentration of lactate.

In recent years, electrochemical biosensors based on FET have been proven to be suitable for detecting biomolecules, because exposed regions of the semiconductor channel can be modified chemically with high-sensitivity functional groups or receptors. The working principle of these biosensors is similar to that of an electrochemical sensor, except that these biosensors use a different recognition layer in which the bio-material is immobilized and used as a transducer [138,139]. The interactions between receptor groups and their targets alter local electric fields, which causes variations in channel conductance, even at low target concentrations. Rim et al. created wearable and highly-sensitive glucose sensors by combining ultra-thin indium oxide (In_2_O_3_) semiconductor-based FETs on the ultra-thin films. [40]. Ultra-thin In_2_O_3_ films, i.e., films that are only 3.5 nm thick, can avoid the intrinsic effects of strain, such as physical strain and peeling from substrates. Figure 6c shows conformal contacted devices on an artificial eye and on human skin with good adhesion, irrespective of the relaxation and tensing of the hand. Conformal contact occurred because the 1.7 μm thickness of the device was less than 1.77 μm, the critical thickness of the device (Figure 6d). Glucose sensing was based on the oxidation of d-glucose by the enzyme glucose oxidase. Figure 6e shows the responses of In_2_O_3_ thin-film FET biosensors upon addition of different glucose concentrations. As the concentration of d-glucose was increased, changes in current responses were detected. Responses showed linear behavior. Also, these In_2_O_3_-based FET biosensors can be used for sensing over the wide range represented by the very low glucose level in tears and the comparatively high glucose level in blood (Figure 6e).

You et al. fabricated a graphene-based FET enzymatic glucose biosensor [140]. This biosensor detected glucose levels by measuring the differential drain-source current and the Dirac point shift of the graphene transistor. Glucose immobilized on the silk film on the graphene FET was oxidized by glucose oxidase, a biorecognition material. When silk is used as the substrate, flexible and biocompatible forms of biosensors can be used in wearable devices. The average sensitivity of the sensor was 2.5 μA/mM measured at *V_ds_* = 100 mV and *V_g_* = 0 V. These organic field-effect transistors (OFETs) possess beneficial properties, such as mechanical flexibility, printability, and low manufacturing cost, making them applicable in wearable sensors [141].

In addition to the types mentioned above, there are also optical biosensors that are based on measuring the changes in the intensity of light and converting light signals into electrical signals that can be recorded in the form of currents or potentials [142,143,144]. Badugu et al. used a colorimetry method to develop a glucose-sensing contact lens [143]. They doped boronic acid-containing fluorophores in the contact lens. Spectral changes in the contact lens occurred due to the interaction between sugar and the fluorophores. However, this sensor required a hand-held device that was used to flash light for non-UV excitation into the eye and measure the intensity of the emissions, making it difficult to provide continuous sensing information.

Ruan et al. fabricated a gelated colloidal crystal array (GCCA)-lens that provided rapid visual detection of analytes through colorimetric determinations of concentrations without additional instruments [144]. These sensor materials were attached to a gas permeable contact lens by gelating a crystalline colloidal array (CCA) embedded in a hydrogel matrix (Figure 6f). This physical gelation method enabled the construction of a hydrogel-based CCA on the irregular surface. When the diols and borate ions in the hydrogel combined with glucose, the volume of the hydrogel was changed, leading to a shift in the diffraction wavelength, which appears as a lens color offset. The change in volume, which is directly proportional to the change in thickness, can explain the shifts in diffracted wavelength by Bragg’s law: λ=2ndsinθ, where *d* is thickness of a given layer in this case, λ is wavelength, *n* is refractive index, and θ is the Bragg angle (during measurements, θ = 90°). Figure 6g shows the diffraction wavelength shift at a relatively lower glucose concentration before the turning point. GCCA-lens’ response the color changes between green and blue within the tear glucose concentration region (0 to 1 mM).

## 3. Multiplexed Sensors

With the development of wearable sensors with excellent flexibility and stretchability, it has become possible to detect various signals, such as temperature, strain, and the presence of ions, and research applications are being expanded. As a result of the additional research, multiplexed sensors have been reported that are capable of simultaneously acquiring and analyzing more information and signals from the natural environment using various single sensors.

**Figure 7:**
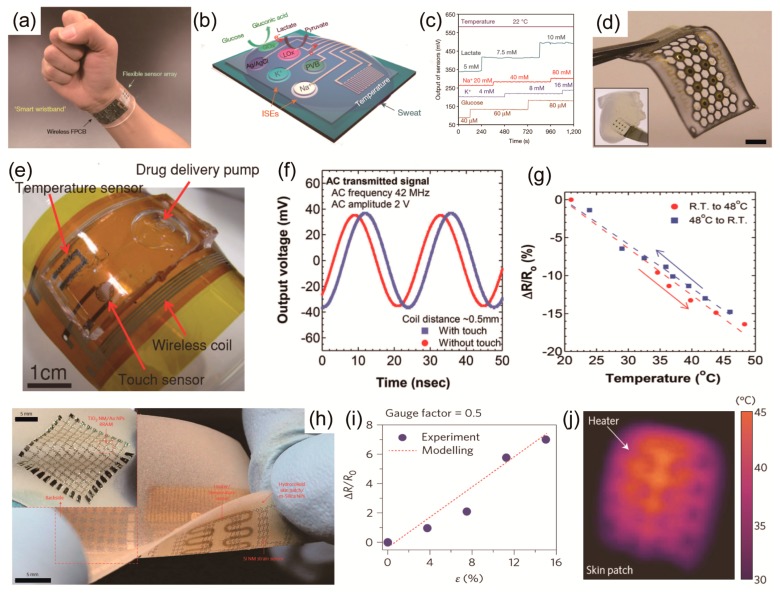
Wearable multiplexed sensors. (**a**) Photograph of a wearable flexible integrated sensing array (FISA) on a subject’s wrist, integrating the multiplexed sweat sensor array and the wireless flexible, printed circuit board (FPCB). (**b**) Schematic of the sensor array (including glucose, lactate, sodium, potassium and temperature sensors) for multiplexed perspiration analysis. GOx and LOx, glucose oxidase and lactate oxidase. (**c**) System-level interference studies of the sensor array. Reprinted with permission from Ref. [38]. Copyright 2014, Nature Publishing Group. (**d**) Optical image of the fabricated ion sensor on a cellular substrate. Ion sensor mounted on a rabbit heart model constructed from agarose gel (inset). (scale bar 2 mm) Reprinted with permission from Ref. [149]. Copyright 2014, John Wiley and Sons. (**e**) Photograph of the fabricated smart band. (**f**) Output AC signal with and without touching. (**g**) Normalized resistance change as a function of temperature. Reprinted with permission from Ref. [62]. Copyright 2014, John Wiley and Sons. (**h**) Photographs of the wearable bio-integrated system. Inset: Wearable 10 × 10 RRAM array on the hydrocolloid side of the patch (scale bars 5 mm). (**i**) Plot of percentage change in resistance versus strain for calculation of the gauge factor (**j**) Temperature distribution measurement of the heater on the skin patch using an infrared camera. Reprinted with permission from Ref. [99]. Copyright 2014, Nature Publishing Group.

Gao et al. developed a wearable, wrist-band sensor that combined a flexible multiplexed sensor and a flexible, printed circuit board (FPCB) [38]. Figure 7a shows a wearable flexible integrated sensing array (FISA) that can simultaneously measure skin temperature during both indoor and outdoor long-term physical activity as well as metabolites and electrolyte panels for human sweat at the same time. By fabricating the flexible sensor on a polyethylene terephthalate (PET) substrate, a stable sensor-skin contact is formed and the FPCB technology uses an easily available integrated circuit to form a component (Figure 7b). This multiplexed sensor can sense a variety of signals through the human skin. For example, exorbitant loss of sodium and potassium in sweat could cause hyponatremia, muscle cramps, hypokalemia, and dehydration [145]; glucose in sweat has been reported to be metabolically related to serum glucose [146]; lactate in sweat can potentially serve as a sensitive marker of pressure ischemia [147], and the temperature of the skin is clinically informative concerning a variety of diseases and skin injuries, such as pressure ulcers [42,148]. In addition, skin temperature measurement is required to compensate and remove the effect of temperature change on readings of chemical sensors through the built-in signal processing device. The performance of each sensor was monitored separately from other analytical solutions. All five sensors are integrated in FISA, and simultaneous system level measurements maintain excellent selectivity when the concentration of each analyte is varied as shown in Figure 7c. The multiplexed sensor was attached to human wrist.

Lee et al. implemented a stretchable multiplexed sensor using a serpentine Au electrode on a cellular substrate with a honeycomb structure. It was confirmed that even though the completed sensor were stretched up to 30% by the Au serpentine electrode and hexagonal honeycomb structure without deformation, as shown in Figure 7d [149]. In addition, Ca^+^, K^+^, and H^+^ ions can be detected in the device.

Another development direction of multiplexed sensors, i.e., a multiplexed sensor for medical purposes, was developed by combining various sensors and drug-delivery devices. These wearable devices are capable of measuring vital signals in real time, and they have the merit of providing more stable and detailed treatment by injecting the appropriate amounts of drugs every hour.

Honda et al. developed an initial type of multiplexed sensor for therapy [62]. A capacitance-type touch sensor and temperature sensor were fabricated on a polyimide (PI) substrate to produce a flexible, multiplexed sensor, as shown in Figure 7e. There is a 1.6 mV amplitude difference and a 44° phase shift with the touch sensor, confirming that the device can successfully detect a touch wirelessly (Figure 7f). Also, the temperature change was measured with a resistor using PEDOT:PSS and CNT (Figure 7g). In addition, a drug chamber using PDMS was fabricated to develop a device capable of putting the drug into the skin using the pressure applied to the chamber.

Son et al. demonstrated wearable bio-integrated systems with optimized performance of data storage, diagnostics, and drug delivery functionality in stretchable formats; their systems were enabled by the integration of bottom-up nanoparticles and top-down nanomembranes (Figure 7h) [99]. In treating Parkinson’s disease, medication is needed to relieve seizures. It is also important to sense the cramps before they become severe so that small amounts of the drug can be infused continuously rather than injecting large amounts at once. In the case of the multiplexed sensor that was developed, a silicon nanowire (SiNW)-based strain sensor measures muscle spasms, puts the results into a memory device, analyzes the results, and heats the heater to inject the drug into the skin at the silica nanoparticle layer, as shown in Figure 7i,j. This system has the advantage of making it possible to perform more stable care by injecting a small amount of drug at that time instead of about drug injection at a time.

In addition, sensors for various purposes have been developed by combining various sensors to sense the surrounding environment and synthesizing the results.

## 4. Wireless Sensors

Wearable sensor technology is essential for monitoring the status of people’s health in an industrial environment [78,79]. Wireless systems on wearable sensors have been studied to wirelessly operate the devices as well as to deliver sensing signals to the external detectors, which leads to continuous monitoring of the internal physiological parameters of the human body [150,151,152,153]. For wireless operation, sensors have been developed that incorporate resonant antennas into resistor, inductor, and capacitor(RLC) circuits, which consist of a resistor (*R*), an inductor (*L*), and a capacitor (*C*) [6]. The resonant antenna is a radiative element in the circuits, and it is used in most real applications. Because the resonance frequency ($f_{r}=1/2\pi \sqrt{L\times C}$) enables the capacitive and inductive reactance to be canceled each other out and wirelessly delivers the maximum amount of energy. The physical properties of capacitance and inductance determine the frequency of the antenna, so that changes in resonance frequency of the antenna coil exhibit the changes of the passive components in the circuits. These concepts enable the electronics to be miniaturized designing wearable sensors that do not require batteries, which makes wireless, skin-mounted devices possible.

In addition, wearable health-monitoring systems integrated with the wireless communication modules can transfer data wirelessly to a distant server in a healthcare facility. In order to do this, the physiological signals measured by the sensors require a two-step communication to deliver the data to the server [154]. The first step is short-range communication. In this step, the sensor transmits the signals to nearby gateway nodes, such as a smartphone, a computer, or a personal digital assistant (PDA). In the second step, the information gathered on the gateway nodes is transmitted to the remote health server through the Internet or a telecommunication system. During the first step, the wearable sensors transmit the data to the gateway nodes over a prolonged period of time through a wired or wireless medium. However, the wired connection may limit the user’s mobility or lead to a failed connection with the devices. Thus, the integration of wearable sensor with wireless technology is preferred, and data-transfer modules, such as Bluetooth, ZigBee, and near-field communication (NFC), have been combined with wearable sensors [155,156,157]. Recently, the remarkable improvements in the designs of flexible and stretchable devices and of packaging technologies have enabled skin-mounted or implantable devices to be integrated with Bluetooth or NFC modules, and this leads to the possibility of accessing remote healthcare via mobile electronic devices.

### 4.1. Resonance Antenna Integrated Sensors

A resonance antenna integrated sensor is based on the electrical RLC resonant circuit, so the sensing results can be monitored by the change in the impedance according to the change in resistance (*R*) or capacitance (*C*). Using this concept, wireless sensors can be demonstrated by the integration of resonance antenna and electronic sensors or capacitive sensors [18,32,158,159]. In the wireless sensors based on electronic sensors, the change in resistance of coils induces the modulation of reflection value (S11) by following the Equation (1),

(1) $$S11=\frac{\omega^{2}M_{12}^{2}}{2Z_{1}Z_{2}+\frac{2\omega^{2}M_{23}^{2}Z_{1}}{Z_{3}}+\omega^{2}M_{12}^{2}}$$

where *M*_xy_ and *Z*_x_ are the coupling coefficient and resistance of reader coil (1), resonate coil (2), and load coil (3), respectively. Their resonance frequency is constant due to no change in capacitance. Furthermore, in the wireless sensors based on capacitive sensors, the detection is realized by the shift of resonance frequency due to the change of capacitance [32,160]. Also, in the case of resonance antennas, it is possible to fabricate antennas with in-plane structures that do not require additional or bulky device chips and energy storage devices [18,32,38,158,159,161]. Therefore, unlike other communication systems, such antennas are fairly easy to miniaturize. In addition, the replacement of antenna electrode materials and the modification of antenna designs make it possible to easily implement wearable and wireless electronics. For these reasons, many groups have conducted extensive studies of wearable and wireless sensors based on resonance antennas, and such sensors have been used in many applications [18,32,158,159,160,162].

**Figure 8:**
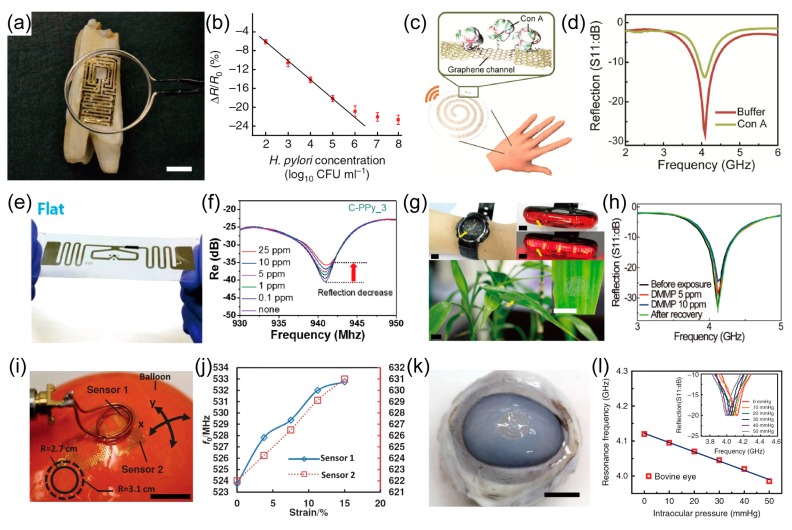
Wearable sensors integrated with resonance antenna. (**a**) Optical image of the graphene-based wireless sensor transferred onto the surface of a tooth (scale bar: 1 cm). (**b**) graphene resistance change versus concentration of H. (**a**,**b**) Reproduced with permission from Ref. [159]. Copyright 2012, Nature Publishing Group; (**c**) Schematic of the biosensor attached to the skin on the back of a human hand; (**d**) Frequency response of the reflection coefficient of the antenna on the plastic substrates after buffer and Con A treatment. (**c**,**d**) Reproduced with permission from Ref. [12]. Copyright 2015, John Wiley and Sons; (**e**) Photographs of the RFID tag sensor; (**f**) Change in the reflectance properties. (**e**,**f**) Reproduced with permission from Ref. [163] Copyright 2016, American Chemical Society. (**g**) Optical photos of wearable gas sensors integrated with resonance antenna transferred onto various substrates (wristwatch, light of bicycle, and a leaf of live plant) (scale bars: 1 cm). (**h**) change in reflection coefficient (S11) of the wireless sensor on the leaf at varied DMMP vapor concentrations (before exposure, 5 ppm of DMMP, 10 ppm of DMMP, and after recovery). (**g**,**h**) Reproduced with permission from Ref. [18]. Copyright 2016, Royal Society of Chemistry. (**i**) Image of a wireless epidermal sensor attached onto the surface of a balloon to simulate measurement of lymphedema. Scale bar, 1 cm. (**j**) Change in resonance frequencies of strain sensors under the expansion of the balloon. (**i**,**j**) Reproduced with permission from Ref. [160]. Copyright 2014, John Wiley and Sons. (**k**) Photograph of the sensor transferred onto the contact lens worn by a bovine eyeball. Scale bar, 1 cm. (**l**) Frequency response of the intraocular pressure sensor on the bovine eye from 5 mmHg to 50 mmHg (Inset: the corresponding reflection coefficients of the sensor). (**k**,**l**) Reproduced with permission from Ref. [32]. Copyright 2017, Nature Publishing Group.

Wearable and wireless sensor platforms based on resonance antennas can be utilized as advanced wearable biosensors [12,160,162]. The wearable biosensors have been studied extensively because they can rapidly detect various biomarkers that represent the status of the human body. Mannoor et al. fabricated wearable and wireless sensors to detect bacterial cells (such as *Escherichia coli* (*E. coli*), *Staphylococcus aureus* (*S. aureus*), and *Helicobacter pylori* (*H. pylori*)) on a silk substrate, as shown in Figure 8a [159]. The sensors are very thin (~50 μm), so they can be attached to objects with rough surfaces, such as enamel. Figure 8b shows a sensor that was fabricated to exhibit high sensitivity with a low detection limit of ~100 bacterial cells. In addition, Kim et al. explored stretchable, transparent, and wireless sensors based on AgNW-graphene hybrid conductors to detect protein (*Concanavalin* A (Con A)), as depicted in Figure 8c [12]. In this platform, the graphene acts as a sensing channel layer to detect Con A, and its resistance is changed by the detection of Con A. As a result, the impedance in the RLC circuit and the reflection coefficient at the resonance frequency are modulated (Figure 8d). Furthermore, the investigated sensors could be stretched up to 20% strain due to the intrinsic stretchability of the AgNW-graphene hybrid structures, so they could be operated wirelessly and stably even on human skin.

Resonance antenna-integrated wearable sensors also have been studied for environmental monitoring [18]. Jun et al. fabricated a wireless and flexible gas sensor based on the carboxyl group functionalized polypyrrole (C-PPy) to detect volatile organic compounds (ammonia and acetic acid), as shown in Figure 8e [163]. By integrating the resonance antenna, the wireless sensors can measure 0.1 part per million (ppm) of ammonia vapor through the difference in the reflection coefficient at the resonance frequency (Figure 8f). In addition, their performances were acceptable during deformations (rolling or twisting) because the devices are so thin. Park et al. explored stretchable and transparent gas sensors based on AgNW-graphene hybrid electrodes [18]. Due to their superior stretchability (stable up to 20% strain), the gas sensors could be mounted onto various objects with large roughness, such as a leaf of a live plant (Figure 8g), and they can monitor dimethyl methylphosphonate (DMMP) vapor at concentrations as low as 5 ppm (Figure 8h). These results offer promising strategies for their use in wireless and wearable electronics, which also is represented to the Internet of Things (IoT).

Wearable sensors integrated with resonance antennas also can measure physical parameters, such as strain and pressure [32,151,160,164,165]. The advanced sensor platforms possess both wearability and wireless communication capability, so they easily can monitor the physical parameters of the human body by merely mounting them on the human body (e.g., skin or tissue) [160]. Huang et al. studied wirelessly operable strain sensors for monitoring health/wellness (e.g., lymphedema or edema), as shown in Figure 8i [160]. They produced wearable and wireless strain sensors by using electrodes and antennas that had serpentine structures. Figure 8j shows the perpendicularly oriented strain sensors on the surface of an expandable balloon that wirelessly measured the biaxial strain of 15% by the shift of their resonance frequency. As shown in Figure 8k, Kim et al. recently presented wearable, transparent, and wireless sensors for detecting intraocular pressure to diagnose glaucoma [32]. The sensors for intraocular pressure require both high stretchability and high transparency, because they must be mounted directly on the eyeball in order to acquire the desired measurements. Thus, the sensors were demonstrated by using the stretchable and transparent AgNW-graphene hybrid structures. In these sensors, the intraocular pressure can be detected by the shift in the resonance frequency since an increase in the intraocular pressure results in a decrease in thickness of the elastomer that is placed between two inductive coils. Subsequently, the decrease in thickness of elastomer causes a change in the capacitance and a shift in the resonance frequency. Based on this sensing principle, the sensors can detect the physiologically relevant range of intraocular pressure, i.e., from 5 to 50 mmHg (Figure 8l).

Based on the results that have been mentioned, diverse wearable sensors integrated with resonance antenna can be miniaturized quite easily. However, these systems have several limitations, including undesired shifts in the resonance frequency or other properties due to the deformation and the short transmitting distance [6,160,166]. Also, the sensor platforms that use resonance antennas require bulky devices for the measurements, so they have limited portability [160,164,165]. Thus, it is essential to demonstrate wearable and wireless sensor platforms that use portable measuring devices (e.g., smartphones).

### 4.2. Bluetooth Integrated Sensors

Since wearable sensor technologies have enhanced people’s quality of life it has become important to exchange information about their physiological condition and motion activities in real time through mutual transfers of information between the devices and monitoring systems [154]. The exchange of information through mutual transfers is measured by the sensors connected in various wireless systems, such as Zigbee [156,167], Bluetooth [168,169,170], Infrared Data Association (IrDA), Wireless Local Area Network (WLAN) [171], and NFC [172]. Among the various wireless transmission technologies, Bluetooth uses a frequency range from 2.4 to 2.485 GHz, which is industrial scientific and medical (ISM) frequency band, and it uses one of the 79 designed channels. It can avoid interference between the systems by using a frequency-hopping method that uses multiple frequencies alternately instead of one frequency. The reasons Bluetooth is getting attention in the field of wireless wearable sensors are (1) it can be used at low cost and low power; (2) the transmission of data can be divided over multiple frequencies because Bluetooth divides the frequency band; and (3) Bluetooth signals, unlike infrared rays, can be transmitted through obstacles, such as walls, so there is no need to visually check the status of the wiring and connections. In addition, it is an indispensable part of the wearable wireless sensor field because many countries around the world use the same technology to comply with Bluetooth standards. Bluetooth is a short-range, RF-based industry standard for the inter-operation of smart devices. It is used extensively for wearable devices at low power and low cost, and it can transmit a maximum distance of 100 m at a maximum data transmission rate of 24 MB/s. Previously, the mutual transfer performance of the Bluetooth was insufficient, and there were many restrictions due to the size of the Bluetooth module for wearable devices. However, due to the recent development of flexible electronic materials, extensive research has been conducted using flexible devices in which wireless communication modules are integrated using a flexible substrate and electrodes. Wearable devices with an integrated Bluetooth module have been studied in recent years because of the miniaturization of the Bluetooth module and the improvement of mutual transfer performance.

**Figure 9:**
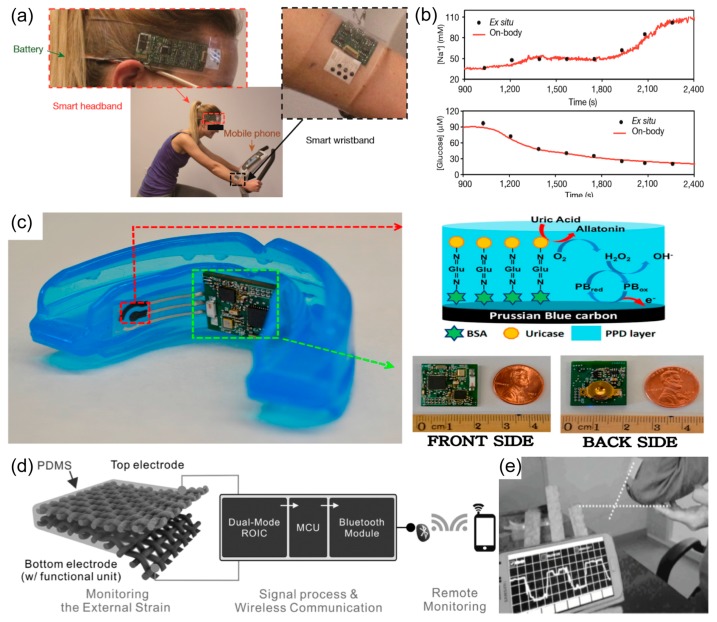
Bluetooth integrated wearable sensors. (**a**) Photographs of wearing a wearable sensor in band types during a stationary cycling; (**b**) Real-time reading data of the sodium and glucose measured with wearable sensors; (**a**,**b**) Reproduced with permission from Ref. [38]. Copyright 2016, Nature Publishing Group; (**c**) Photographs of the wireless SUA biosensor module with Bluetooth and the operation principles of mouthguard biosensor; (**c**) Reproduced with permission from Ref. [29]. Copyright 2015, Elsevier; (**d**) Schematic illustration of a flexible textile-based strain sensor with wireless monitoring system; (**e**) Photographs of the flexible textile-based strain sensor with Bluetooth module and real-time operation via wireless communication with mobile phone. (**d**,**e**) Reproduced with permission from Ref. [83]. Copyright 2016, John Wiley and Sons.

As described by Gao. et al., a wearable, multi-sensor, which enables the detection of electrolytes, such as sodium and potassium ions, glucose, and lactate, can be integrated with the Bluetooth module to wirelessly monitor the physiological information contained in people’s sweat [38]. This wireless sensor enables users to check an individual’s health condition in real time through a smartphone. In addition, it is capable of storing sensing data through mutual transfer between mobile devices and the Bluetooth module. As shown in Figure 9a, this sensor can be used to measure people’s sweat in real time while a subject is wearing a ‘smart headband’ and a ‘smart wristband’ during stationary cycling. This sensor is fabricated to detect a human sweat to extract the complex information from sweat. It is also a fully integrated multiplex detection system, which is sensed by the potential difference of the electrolyte through mechanisms such as biomarker partitioning and passive or active partitioning [173]. These real-time sensing results of the sodium and glucose sensor measured on the body are comparable to the ex situ results (Figure 9b). To measure the uric acid in people’s saliva(SUA), Kim et al. fabricated a wirelessly-operable mouth-guard biosensor in which both the sensor array (red box) and the module (green box) were loaded, as shown in the Figure 9c [29]. To use the device inserted in the body for a long term, they used Bluetooth Low Energy (BLE) technology, which enable them to reduce the power consumption [170,174]. Choi et al. fabricated a wearable gas sensor with the capability of Bluetooth communication. The sensor was fabricated on a polyimide film by using graphene oxide, and it detected chemical substances, including H_2_S, C_2_H_5_OH, and H_2_ [175]. The integration of the Bluetooth module with the sensor can be used for wirelessly monitoring the individual’s environment and status. Lee et al. fabricated a flexible textile strain sensor by using carbon hybrid materials (carbon nanotube/reduced graphene oxide) as electrodes and ZnO nanowires as the active channel layer. The sensor was integrated with a Bluetooth module for real-time detection of the strain variation due to the subject’s movements (Figure 9d). Figure 9e shows that the sensor was attached to the subject’s clothes, and the sensing results were displayed on a smartphone when the subject bends an arm. These wearable strain sensors integrated with Bluetooth modules are expected to be used in the healthcare field as well as in the sportswear, automotive, aeronautic, medical applications, industrial safety, and other fields [83].

### 4.3. Near Field Communication (NFC) Integrated Sensors

NFC is a non-contact, near field wireless communication (operation distance of ~20 cm), and it facilitates the simultaneous transfer of both power and data. It is a next-generation communication technology for wearable electronics because of its (1) compatibility with mobile electronic devices; (2) relatively high security due to its short operational range; (3) possibility of miniaturization of the chip to just a few millimeters; and (4) operation in the passive mode without a battery [154]. The NFC connected with wearable sensors operates in reader/writer mode. In this operational mode, a smartphone initiates the communication as an active device and can both read and write to an NFC tag, which consists of a chip, a sensor, an antenna, and other components. Recently, the advances of stretchable packaging technology have resulted in the production of NFC-enabled sensors in a wearable and skin-attachable form by enhancing the mechanical softness of the entire system [157,176,177,178]. When mounted on a person’s body, these sensors enable the continuous monitoring of the physiological signals and simultaneously digitize the detection results in mobile electronics.

**Figure 10:**
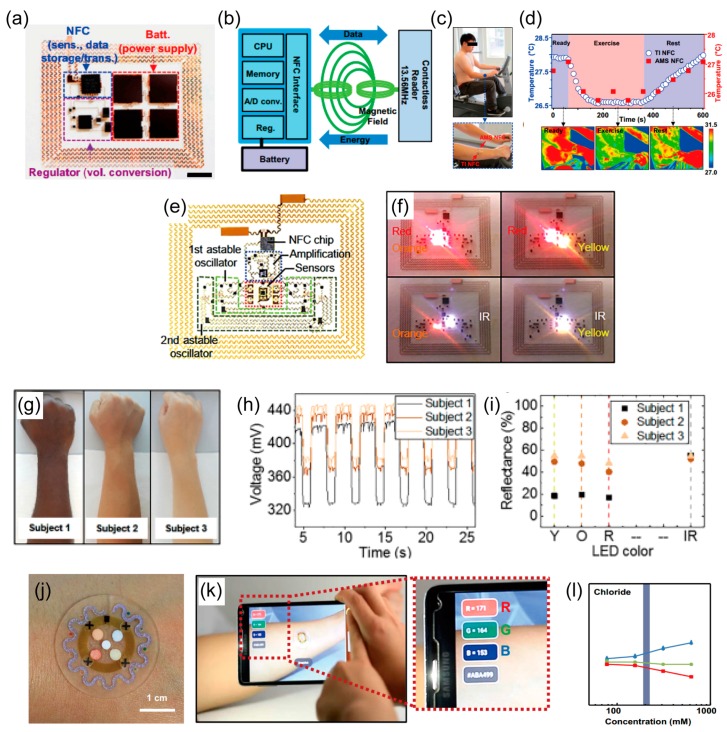
Near field communication (NFC)-enabled wearable sensor. (**a**) Image of the NFC-enabled wearable sensor system composed of the NFC devices, batteries, and a power regulator. Scale bar, 5 mm; (**b**) Schematic illustration of the entire operation system; (**c**) Photo images of the NFC-enabled wearable sensor attached onto the human skin during exercise; (**d**) Temperature data and IR images at the stage of the before, during, and after exercise (which correspond to the “ready”, “exercise”, “rest”) recorded by using NFC-enabled wearable sensor with a battery module (AMS NFC, red squares) and with the battery-integrated system (TI NFC, open circle). (**a**–**d**) Reproduced with permission from Ref. [176]. Copyright 2016, National Academy of Sciences; (**e**) Image of a NFC-enabled wearable sensor system including four pulsed LEDs (red, IR, orange, and yellow), two oscillators, amplifier and sensors; (**f**) Photo images of the NFC-enabled wearable sensor system during operation; (**g**) Images of subjects with different skin colors; (**h**,**i**) Wirelessly measured data and calculated reflectance value of the different skin colors. (**e**–**i**) Reproduced with permission from Ref. [157]. Copyright 2016, American Association for the Advancement of Science; (**j**) Optical image of a fabricated device mounted on the forearm; (**k**) Pictures demonstrating NFC between a sweat monitoring device and a smartphone to launch software for image capture and analysis; (**l**) Standard calibration curves between normalized %RGB value and concentration of markers for quantitative analysis (error bars show s.d. (*N* = 3)). (**j**–**l**) Reproduced with permission from Ref. [177]. Copyright 2016, American Association for the Advancement of Science.

The power supply has been an issue in the development of body-integrated electronics. Even though many researchers have reported impressive results in the fields of energy generation and storage, the limitation of the soft mechanical properties has hindered the practical use of devices attached to the human body. Lee et al. reported the integration of an NFC chip with a stretchable energy storage system composed of a photovoltaic solar cell and rechargeable lithium-ion batteries as shown in Figure 10a,b [176]. The components of the device were interconnected with the fractal-designed electrodes, and the entire system was encapsulated by using a low-modulus elastomer, i.e., the moduli of the core and the shell were ~3 and ~60 kPa, respectively, in order to provide stretchabiltiy and environmental protection. For example, both the solar cell and the battery can endure ~55% and ~45% elongation, which is almost twice as elastic as human skin. In addition to the battery system, the device can be operated wirelessly by using an NFC chip. The NFC device with built-in temperature sensor can be integrated with a power supply in the stretchable system, and this skin-attachable, wireless sensor can deliver sensing data through the NFC to the smartphone for use in analyzing skin thermography of the human body (Figure 10c,d). Even though it may be possible to fabricate a wireless sensor that can work interactively with a smartphone, there is a limitation to the applicability of various sensors. For example, they only use the built-in sensor in the NFC chip. In addition, the use of an external battery results in enlarging the size of the device.

To operate the external sensor without the external power supply, Kim et al. reported a battery-free, wireless optoelectronic system [157]. The NFC technology allows power to be delivered to the devices by using a magnetic inductive coupling, and the sensing results are extracted and displayed on a smartphone. As a demonstration, they fabricated a photodetector to measure the optical properties of the human skin and used multi-colored light-emitting diodes (LEDs) to present the sensing results (Figure 10e). The devices were connected externally to the NFC chip and integrated with the fractal designed interconnection in the elastomer for the conformal contact on the skin. The multi-colored LEDs (Figure 10f) were designed to emit different wavelengths wirelessly, depending on the reflectance of the targeted color. Figure 10g shows the different skin colors of the three subjects, and the NFC-enabled photo detectors wirelessly measured the differences in the colors (Figure 10h). Figure 10i shows that the LEDs emitted yellow, orange, and red colors, corresponding to subjects 1, 2, and 3, respectively.

Koh et al. reported an NFC-operable sweat sensor that could detect the concentrations of chloride, lactate, and glucose, as well as pH, as shown in Figure 10j [177]. In order to harvest the sweat from the human skin in a microfluidic system, they used an artificial pore system that included a perforated PI membrane to mimic sweat glands. When the sweat entered the microfluidic channel, the enzyme changed the hydration status of the analytes, and the dyes in the water changed their colors. For example, the anhydrous cobalt chloride chelated with water was converted to hexahydrate cobalt chloride, and the color changed from deep blue (*λ*_max_ = 657 nm) to pale purple (*λ*_max_ = 511 nm). In addition, to detect the concentration of the glucose, they used glucose oxidase for enzymatic reaction which is capable of oxidation of glucose and reduction of oxygen. This reaction produced hydrogen peroxide, and it enables the iodide to oxidize to iodine, which makes color change from yellow to brown. The concentration of the target materials can be analyzed quantitatively by ultraviolet (UV)-visible spectroscopy. After fabrication of the microfluidic sensor that indicated the detection results as colorimetric responses, the sensor was integrated with the NFC chips to record color changes and to convert the colors into the exact red, green, and blue (RGB) colors of the smartphone display. Figure 10k shows the devices mounted on the human skin and operating stably while displaying RGB values. After wirelessly recording the images, the RGB colors, which can be exported as percentages in the RGB format, can be expressed as analyte concentrations, as shown in Figure 10l. This wireless sweat sensor can detect changes of 0.5 pH units and 0.2, 0.3 and 0.1 mM chloride, and lactate and glucose concentration, respectively, and they correspond to a 1% change in R channel of RGB images. Similarly, Akira et al. reported NFC-operable UV dosimeters that included photo-activators (PPDP-TF) and two types of color changeable dyes (CVL and Congo red, which respond to UV-A and UV-B, respectively) [178]. After absorption of the UV photons, the photosensitive activator generates radical species, which enables colorless dyes to become brightly colored. After exposure to the UV radiation, the smartphone enables the analysis of the RGB color from the UV dosimeters. These colorimetric sensing approaches are compatible with the NFC-enabled smartphone, and they also are applicable other types of environmental constituents, such as pollution or toxic gases.

## 5. Conclusions

In this review, we summarized recent advances in wearable electronic devices, including smart sensors and wireless systems, for real-life applications in monitoring people’s activities and their personal healthcare. Smart sensors that have various configurations, such as piezo-resistive, piezo-electric, and capacitive types, can monitor various human physiological signals. The recently developed smart sensors are flexible and stretchable so that they can resist external deformation generated by human activities. These smart sensors can be classified into individual sensors that measure only one signal and multiplexed sensors that measure two or more signals, depending on the number of physiological signals being measured. Also, the individual sensors can be classified as physical sensors, which are those that measure temperature, pressure, and strain, chemical sensors, which are those that measure gases and ions, and biosensors, depending on the type of physiological signals being measured. Currently, the research efforts are focused more on the development of multiplexed sensors, which enable multiple detections, such as temperature, pressure, and strain, for precise monitoring of healthcare. Using smart sensors, the condition of a person’s health can be monitored in real time, and the diagnostic results can be transported to control and processing units. Most of the recently reported studies related to wearable electronic devices used wireless systems to transmit the diagnostic data because wires can hinder the user’s mobility, make the device feel uncomfortable on the body, and increase the risk of system failure. As mentioned earlier, these wireless systems can be classified into Bluetooth, NFC, and resonance antenna systems, according to the driving principle. Someday, these wearable electronic devices will allow us to monitor people’s health status in real time with a smartphone and make telemedicine a reality, including injecting medications and scheduling appointments wirelessly.

Despite the progress described above, many challenges still remain before the implementation of wearable electronic devices in practical applications. For example, high performance sensors are affected by the external noisy signals generated by the human motion. To weaken the disturbance from these external noisy signals, cost-effective packaging and manufacturing methods should be accompanied with the fabrication of wearable electronic devices. Micro Electro Mechanical Systems (MEMS) technology is widely used for manufacturing, but it needs expensive production equipment. Recently, new technologies, such as 3D-printing technology [179,180,181], are capable of reducing the fabrication costs and enabling mass production contrast to the traditional step-by-step manufacturing technology. Moreover, conformal attachment of strain and pressure sensors on the human body is also essential for more accurate measurements of the human motion. For this purpose, the intermediate adhesive layers [4,5,86,182] such as microhair adhesive structures [183,184,185,186] can be integrated with the strain and pressure sensors for enhanced contact efficiency. Also, designing low power consuming wearable electronic devices has always been an exciting but challenging issue. To provide the required operating power of wearable electronic devices, sensing devices that are self-powered have been introduced [187,188,189], although there still are challenging issues regarding the extended sustainability of the flexible power source. We believe that these challenges will be gradually solved, leading to such wearable electronic devices, which allow us to monitor our health status in real-time with a smart phone, and doctors to practice telemedicine simply from scheduling an appointment even to wireless drug injections.

## Figures and Tables

**Table 1:** Summary of the wearable sensors.

Sensor Type	Sensing Element	Substrate	Electrode	Active Material	Working Voltage	Sensing Range	Sensitivity	Flexibility	Stretchability	Reliability	Reference
Temperature	Human hand temperature	PDMS or thin PVA	Au	Au	0.6 V	20~150 °C	1.9 Ohm K^−1^	-	100%	-	[42]
Temperature	Temperature	PDMS	AgNW	Graphene	10 V	30~100 °C	Nonlinear resistance response	-	50%	100	[31]
Temperature	Endothelial layer	Polyester fabric strip coated with PDMS	Au	Platinum (Pt)	0.8 V	0~120 °C	2.7 Ohm K^−1^	0.01%	-	-	[54]
pressure	Intraocular pressure (IOP)	Parylene	graphene-AgNW	ecoflex (capacitive-type)	-	5~150 mmHg	-	Bending radius: 3.1 μm	25%	10,000 cycles with 25% strain	[32]
Pressure	Cutaneous pressure	Ecoflex	Au	Lead zirconate titanate (PbZr0.52Ti0.48O3, PZT)	VG: 3.5 V VD: 0.1 V	0.005~10 Pa	1.36 μA Pa^−1^	14.8 mm bending radius	30%	1000	[3]
Pressure	Touch	PET or Polyurethane	Conductive carbon fabric	CNT/PDMS porous	0.1 V	0.25~100 kPa	-	30 mm bending radius	30%	10	[39]
Pressure	Artery wrist pulse or acoustic vibrations	PDMS	Au	Au NWs	1.5 V	13 Pa~50 kPa	1.14 kPa^−1^	30 mm bending radius	25%	50,000	[36]
Pressure	Pressure	PDMS and Epoxy (SU8)	Au or AgNW/graphene	Graphene	VG: 25 V VD: 0.1 V	250 Pa~3 Mpa	2.05 × 10^−4^ kPa^−1^ (below 500 kPa), 9.43 × 10^−6^ kPa^−1^ (above 500 kPa)	-	-	1000	[33]
Strain	Facial expressions	PDMS	Polyurethane-PEDOT:PSS	Single-walled carbon nanotubes	1 V	1.6~3.6%, 10~100%	62 (Δ*R*/*R*)/*ε*	3.60%	100%	Minimum 50 cycles for bending, 1000 cycles for stretching	[34]
Strain	Heartbeats	PDMS	Graphene woven fabrics	Graphene woven fabrics	1 V	under 0.2~30%	1000 (Δ*R*/*R*)/*ε*	-	30%	-	[86]
Strain	Stretch and pressure	PDMS	Eutectic Gallium Indium	Single-walled carbon nanotubes-Ecoflex	-	~150%	0.004 (Δ*C*/*C*)/*ε*	-	150%	-	[69]
Strain	Joint movement (bending)	Silicon elastomer (Dragonskin 10 + Thi-Vex silicon thickener + Slo-jo platinum silicon cure retarder)	Silver wire	Ionic fluid - silicon elastomer	AC 5 V, 50~200 Hz	~700%	0.348 (Δ*C*/*C*)/*ε*	-	700%	minimum 20 cycles for stretching	[92]
Strain	Bending	PDMS coated Polystyrene	Silver paste	ZnSnO_3_ nanowires	1.2 V	~0.33%	3740 (Δ*I*/*I*)/*ε*	0.33%	No stretchability	-	[95]
Strain	Lymphedema	Silicon rubber (Solaris)	Cu	Cu-PI (capacitive-type)	-	0~30%	-	-	30%	-	[160]
Strain	Strain	PET textile	Carbon nanotube/reduced Graphene oxide	ZnO Nanowire	-	Sensor limits of bacterium/μL	-	3~5%	-	100 bending cycles	[83]
Gas	O_2_ (oxygen)	Porous PTFE (porous polytetrafluoroethylene)	Au	High-purity 1-butyl-1-methylpyrrolidinium bis (trifluoro-methylsulfonyl)imide	−1.4V	0~21%	0.48 uA/%	Can be bent either convex or concave	-	-	[117]
Gas	NO_2_ (nitrogen dioxide)	PES (polyethersulfone)	Cr/Au	Graphene	30 V	0.5 to 40 ppm	∆*R*/*R*_0_ = −40% (at 40 ppm N2)	∆*R*/*R*_0_ = 5% under a bending strain of 1.4%	-	-	[37]
Gas	NO_2_ (nitrogen dioxide)	Paper	Au	NaNO2 treated PbS CQD	4.1 V	0.5 to 50 ppm	0.41/ppm	Bending angle of 70°	-	7% decrease in response under 5000 cyclic bending tests (Bending angle of 50°)	[114]
Gas	Volatile compouds (ammonia, acetic acid)	Plastic substrate	Cr/Au	Polypyrrole (Ppy) (functionalized by carboxyl group)	−0.1~0.1 V	0.1~100 ppm	-	Bending angle: 15°	-	-	[163]
Gas	Dimethyl methylphosphonate (DMMP)	PI or PDMS or parylene	Graphene-AgNW	Graphene (functionalized by polypyrrole (Ppy))	0.1 V	5~25 ppm	-	-	20%	1000 cycles with 5% strain	[18]
Gas	H_2_S, C_2_H_5_OH, H_2_	PI	Ti/Au	Graphene oxide	-	Graphene limits of bacterium/μL	Toluene, Acetone, CO Ethanol at 20 PPM, Acetone at 20 ppm, H2S, H2 at 5–20 ppm	A 30° bending angle	-	104 bending cycles	[175]
Bio	Protein (Concanavalin A, Con A)	PET or PDMS or parylene	Graphene-AgNW	Graphene (functionalized by mannosyl-pyrene)	0.1 V	1 mg/mL	-	Bending radius: 27 μm (bending strain: 2.59%)	20%	-	[12]
Bio	Bacteria (*E. coli*, *S. aureus*, *H. pylori*)	Silk fibroin film	Cr/Au	graphene (functionalized by antimicrobial peptides (AMPs))	-	Detection limits of bacterium/μL	100~10^8^ CFU/mL	-	-	-	[159]
Bio	Lactate	Temporary transfer tatto paper, GORETEX	Ag/AgCl, conductive carbon	lactate oxidase(LO_x_)	0.05 V	1 mM to 25 mM	644.2 nA/mM at RT, 0.916 μA/mM at 37 °C	Bending angle of 90°	stretched at ~10%	The bending/stretching test: 10 times	[136]
Bio	D-glucose	Polyimide film (2 μm)	Cr/Au	In_2_O_3_, glucose oxidase	0.2~0.8 V	100 μM to 4 mM	-	Bending radius of 837 (0.078~0.082%)	-	-	[40]
Bio	Glucose	PDMS, Polyimide, Parylene	Graphene/AgNW	Graphene, glucose oxidase	0.1 V	1 μM~10 mM	-	-	∆R < 6% at 25% tensile strain (5000 cycles)	∆R ~20% at 10,000 cycles of stretching	[32]
Bio	Glucose, Lactate	PDMS	-	Enzyme and chromogenic reagent	-	Glucose: 0~25 mM Lactage: 0~100 mM	-	5 cm bending radius	Strain 30%	-	[177]
Bio	Salivary Uric acid	PET	Ag/AgCl	Prussian-blue-graphite	-	Mouthguard limits of bacterium/μL	2.45 μA/mM	-	-	-	[29]
Ion	Chloride, H^+^	PDMS	-	Enzyme and chromogenic reagent	-	Chloride: 0~625 μM pH: 5.0~8.5	-	5 cm bending radius	Strain 30%	-	[177]
Ion	Na^+^, K^+^	Textile	Ecoflex-containing Ag/AgCl ink, MWCNT	Multi-walled carbon nanotubes (functionalized by carboxylic acid)	-	Physiological range of human sweat (10~110 × 10^−^^3^ M NaCl, 1~8 × 10^−^^3^ M KCl)	54.1 ± 1.5 mV/log [Na^+^], 56.9 ± 1.6 mV/log [K^+^]	Bending angle of 180°	100 % (uniaxial stretching)	No sensitivity degradation at linear strain of 75% during 60 min at a speed of 1 mm/s	[122]
Ion	Na^+^, K^+^	PET (polyethylene terephthalate)	Cr/Au, Ag/AgCl	Multi-walled carbon nanotubes	3.7 V	1 mM KCl and 10 mM NaCl (working condition)	62.5 mV/log [Na^+^], 59.5 mV/log [K^+^]	Radius of curvature 1.5 cm	-	No sensitivity degradation by bending radius of 1.5 cm over 60 cycles	[38]
Ion	H^+^	Poly (methylmethacrylate)	Graphite	Graphene	−0.4~0.4 V	pH 5~8	17 mV/pH	Radius of curvature: 1.2 cm	0.04	No significant change in the electrical response (mobility values remained constant) as a result of bending to radii of curvature as small as 0.7 cm (estimated bending-induced strain: 0.6%)	[126]
Ion	Ca^2+^, H^+^	PET (polyethylene terephthalate)	Ag/AgCl reference electrode	Polyaniline (PANI)	-	Typical physiological [Ca^2+^] variations (e.g., 1 mM to 0.5 mM), pH 4~7	32.7 mV/log [Ca^2+^], 62.5 mV/pH	-	-	-	[127]
